# Geographical variation in *Plasmodium vivax* relapse

**DOI:** 10.1186/1475-2875-13-144

**Published:** 2014-04-15

**Authors:** Katherine E Battle, Markku S Karhunen, Samir Bhatt, Peter W Gething, Rosalind E Howes, Nick Golding, Thomas P Van Boeckel, Jane P Messina, G Dennis Shanks, David L Smith, J Kevin Baird, Simon I Hay

**Affiliations:** 1 Department of Zoology, Spatial Ecology and Epidemiology Group, Tinbergen Building, University of Oxford, South Parks Road, Oxford, UK; 2 Department of Ecology and Evolutionary Biology, Princeton University, Princeton, NJ, USA; 3 Australian Army Malaria Institute, Enoggera, Queensland, Australia; 4 Department of Epidemiology and Malaria Research Institute, Johns Hopkins Bloomberg School of Public Health, Baltimore, MD, USA; 5 Fogarty International Center, National Institutes of Health, Bethesda, MD, USA; 6 Eijkman-Oxford Clinical Research Unit, Jalan Diponegoro No 69, Jakarta, Indonesia; 7 Nuffield Department of Medicine, Centre for Tropical Medicine, University of Oxford, Oxford, UK

**Keywords:** Malaria, *Plasmodium vivax*, Map, Relapse, Periodicity, Recurrence, Recrudescence, Strain

## Abstract

**Background:**

*Plasmodium vivax* has the widest geographic distribution of the human malaria parasites and nearly 2.5 billion people live at risk of infection. The control of *P. vivax* in individuals and populations is complicated by its ability to relapse weeks to months after initial infection. Strains of *P. vivax* from different geographical areas are thought to exhibit varied relapse timings. In tropical regions strains relapse quickly (three to six weeks), whereas those in temperate regions do so more slowly (six to twelve months), but no comprehensive assessment of evidence has been conducted. Here observed patterns of relapse periodicity are used to generate predictions of relapse incidence within geographic regions representative of varying parasite transmission.

**Methods:**

A global review of reports of *P. vivax* relapse in patients not treated with a radical cure was conducted. Records of time to first *P. vivax* relapse were positioned by geographic origin relative to expert opinion regions of relapse behaviour and epidemiological zones. Mixed-effects meta-analysis was conducted to determine which geographic classification best described the data, such that a description of the pattern of relapse periodicity within each region could be described. Model outputs of incidence and mean time to relapse were mapped to illustrate the global variation in relapse.

**Results:**

Differences in relapse periodicity were best described by a historical geographic classification system used to describe malaria transmission zones based on areas sharing zoological and ecological features. Maps of incidence and time to relapse showed high relapse frequency to be predominant in tropical regions and prolonged relapse in temperate areas.

**Conclusions:**

The results indicate that relapse periodicity varies systematically by geographic region and are categorized by nine global regions characterized by similar malaria transmission dynamics. This indicates that relapse may be an adaptation evolved to exploit seasonal changes in vector survival and therefore optimize transmission. Geographic patterns in *P. vivax* relapse are important to clinicians treating individual infections, epidemiologists trying to infer *P. vivax* burden, and public health officials trying to control and eliminate the disease in human populations.

## Background

Malaria is a significant global public health problem and the greatest burden of disease is found in the world’s poorest countries [1]. The majority of malaria morbidity and mortality is caused by two of the five species of *Plasmodium* that naturally infect humans, *Plasmodium falciparum* and *Plasmodium vivax*. The broader global distribution of *P. vivax* relative to *P. falciparum* puts an estimated 2.5 billion people at risk for endemic vivax malaria [2,3]. An increasing body of evidence has shown that *P. vivax* should no longer be thought of as a benign and rarely fatal disease [4-9], but instead as being capable of causing severe disease and death, particularly in pregnant women and small children [9-12].

*Plasmodium vivax* is epidemiologically and biologically different to *P. falciparum* and it is not, therefore, appropriate to assume that control methods developed for falciparum malaria are directly transferable to *P. vivax*[13-16]. Biological features of *P. vivax* that distinguish it from *P. falciparum* also present unique challenges to the control of the parasite [17-19]; in elimination settings, *P. vivax* is often the “last parasite standing” following *P. falciparum* elimination [20,21]. *Plasmodium vivax* gametocytes are present earlier in the progression of a primary or recrudescent infection compared to *P. falciparum*[17,22], such that the majority of patients have sufficient gametocytaemia to allow for transmission before diagnosis or treatment may occur [23-25]. In addition, *P. vivax* gametocytes are transmitted more efficiently to *Anopheles* mosquito vectors than those of *P. falciparum* and are transmissible at lower parasite densities [18,26,27]. Within the mosquito, *P. vivax* sporozoites develop faster than *P. falciparum* at equivalent temperatures, which contributes to its exploitation of a wider geographic range [28].

**Figure 1:**
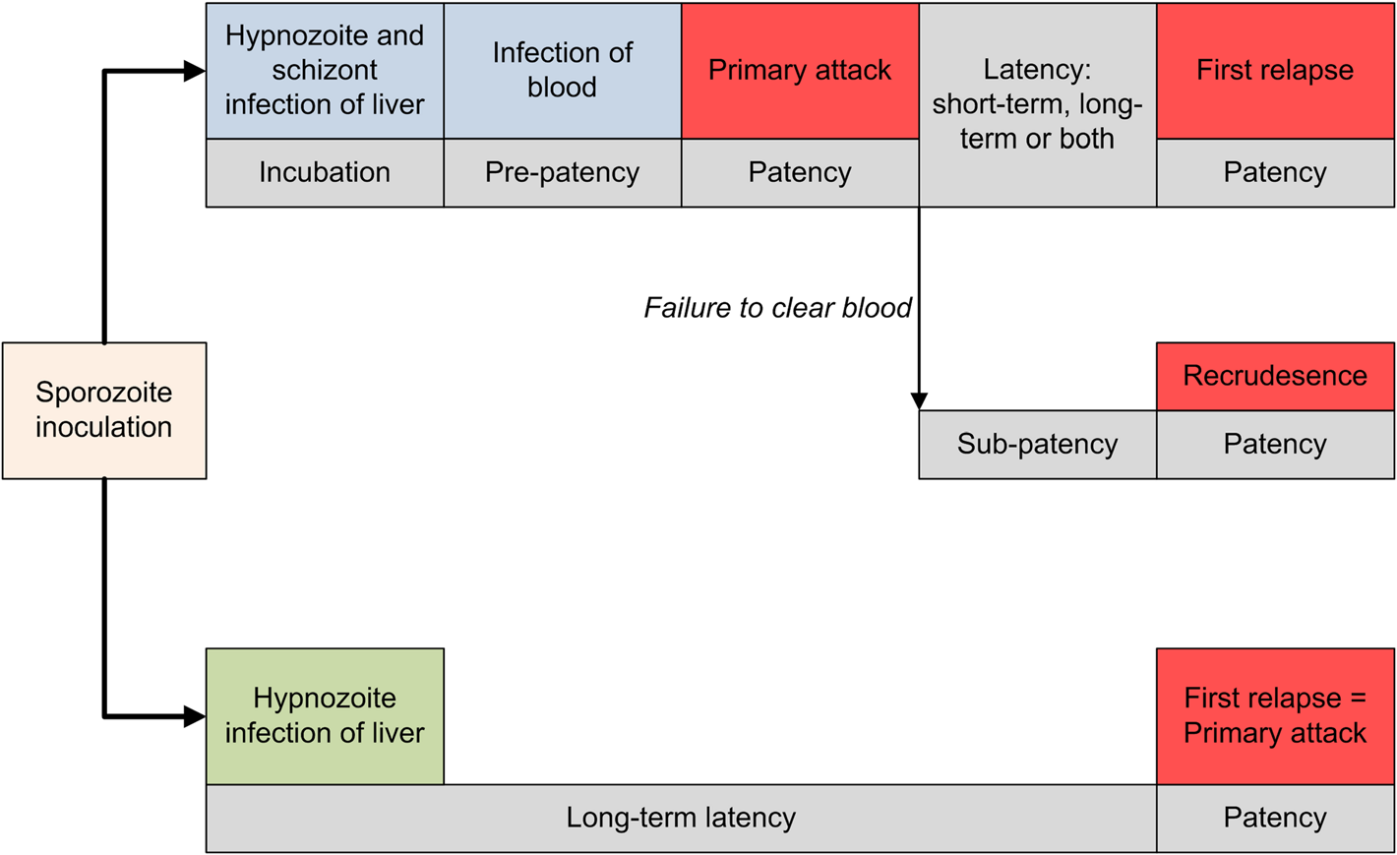
**Pathways to infection of blood and clinical attacks in *Plasmodium vivax *** **malaria.**

Perhaps the most epidemiologically important feature of *P. vivax* biology is its ability to relapse in the weeks and months following a primary parasitaemia via a dormant liver stage known as the hypnozoite [29-31]. This potential for long-term latency provides the obvious advantage of safe harbour during cold winter months when circulation in blood creates potential host immune system dangers without the benefit or opportunity for onward transmission. Therefore the term “infection” has various meanings for *P. vivax*. Infection may refer to the introduction and presence of parasites in the body, but with *P. vivax*, unlike *P. falciparum*, this can also refer to a symptom-less latent infection. The origin of renewed parasitaemia following a primary vivax infection or a “recurrence” is also ambiguous; it could be due to a hypnozoite-triggered relapse, a resurgence of erythrocytic parasites as a recrudescence, or an entirely new reinfection. See Figure 1 and Table 1 for a description of the pathways between types of infection and attack, and distinctions in terminology. The hypnozoite fundamentally distinguishes *P. vivax* from *P. falciparum* in almost every important biological, epidemiological, clinical, and public health respect.

**Table 1:** **Glossary of terms relevant to *Plasmodium vivax *** **relapse**

**Term**	**Definition**
**Infection**	Presence of parasites in any of its forms of incubation, prepatency, patency, subpatency or latency.
**Incubation**	The period between inoculation of sporozoites and release of merozoites into the blood stream (primary exo-erythrocytic cycle).
**Prepatency**	The period prior to a primary attack where asexual parasites in the blood are both not detectable and asymptomatic, though present.
**Patency**	The period of clinical attack with demonstration of asexual parasites in blood as the cause of illness.
**Subpatency**	The period following a primary attack where asexual parasites are both not detectable and asymptomatic, though present.
**Recrudescence**	An event following sub-patency when parasites are both demonstrated to be present and the cause of another clinical attack or asymptomatic patency.
**Latency**	The period between a primary attack and a relapse; in some strains also the period between inoculation of sporozoites and the occurrence of a patent primary attack, typically six months or more.
**Relapse**	Patent asexual parasitaemia originating from activation of latent hypnozoites.
**Re**-**infection**	Patency by asexual blood stage parasites deriving from a new inoculation of sporozoites.
**Recurrence**	A newly patent parasitaemia occurring at any point after clearance of sub-patency of a primary parasitaemia where the origin is not known with certainty as being a reinfection, recrudescence or relapse.

Definitions are presented in chronological order of events.

The hypnozoite stage in the life cycle of *P. vivax* and the potential for relapse makes chemical therapies that target only the blood stage of infection ineffective as a radical cure. The 8-aminoquinolines are the only class of drugs known to have activity on the hypnozoite parasite [32-34]. Primaquine therapy, the only currently licensed radical cure, comes with caveats that add to the challenge of controlling the parasite to the point of elimination. Primaquine is associated with potentially fatal haemolysis in individuals with glucose-6-phosphate dehydrogenase (G6PD) deficiency [32,35,36] and is contra-indicated in pregnant women because of the risk of acute haemolytic anaemia in the foetus of unknown G6PD status [37]. The hypnozoite stage and the paucity of therapy for safe and effective treatment render vivax malaria an exceedingly difficult challenge for clinicians and those responsible for the control of endemic malaria. Relapse also has critical implications for understanding epidemiological metrics such as the basic reproduction number and force of infection, obtained from prevalence rates derived from malariometric surveys and cartographic studies that form a central part in elimination scenario planning [38,39].

It has long been known that there is significant geographical variation in the rate at which a “strain” of *P. vivax* relapses [40-42]. Temperate and subtropical strains often exhibit either a long incubation or latent period (Figure 1) of around eight to ten months. Tropical strains are characterized by short incubation times and short latency (approximately three to six weeks) [43]. Incubation period refers to the time from sporozoite inoculation (the mosquito bite) to the primary blood-stage infection. The latent period describes the time from the primary attack to relapse. How hypnozoite relapse is triggered, and the source of this phenotypic variation, is unresolved [44]. One theory is that the mechanism is an adaptive trait of the parasite to sequester or “hibernate” during times when climatic conditions would be inhospitable to the parasite’s anopheline mosquito vectors [45-47]. Another is that latent hypnozoites are activated by a systemic febrile illness, explaining the large number of *P. vivax* relapses that follow *P. falciparum* infections [47-49]. These hypotheses need not be mutually exclusive.

Regardless of the triggering mechanism and aetiology of relapse, evidence from both controlled experimental and natural settings indicate considerable geographical variation in the timing of relapse. Although the historical perception has been that frequent relapsing strains originate from the tropics and long-latency strains from temperate regions [31], it does not sufficiently describe the observed variation in relapsing phenotypes. This binary classification conflicts with evidence of long-latency strains in tropical regions in the Americas, for instance. Coatney *et al*. [43] described in 1971 that there were three patterns of relapse. These included the Chesson strain of New Guinea-South Pacific which exhibits a short incubation period (seven to fourteen days), followed by regular re-invasions of the bloodstream within approximately three weeks after the primary infection and may continue to relapse for more than 18 months without a radical cure of the hypnozoite stage. The St Elizabeth strain from southern USA has a similar incubation period to the Chesson strain, but hypnozoites remain quiescent for several months following the primary infection before relapsing at regular intervals of three to four weeks for up to two years [43]. A third variety, such as the strain once found in the Netherlands [42], has a long period of incubation (around eight months) before the primary clinical episode followed by frequent relapses (the lower arm of Figure 1). This three-type classification likely oversimplifies the degree of variation in *P. vivax* relapse periodicity and offers limited information regarding the geographic origin of the described phenotypes. Furthermore, the majority of *P. vivax* strains have short incubation periods and the greatest difference lies in the latency period from primary attack to first relapse.

**Figure 2:**
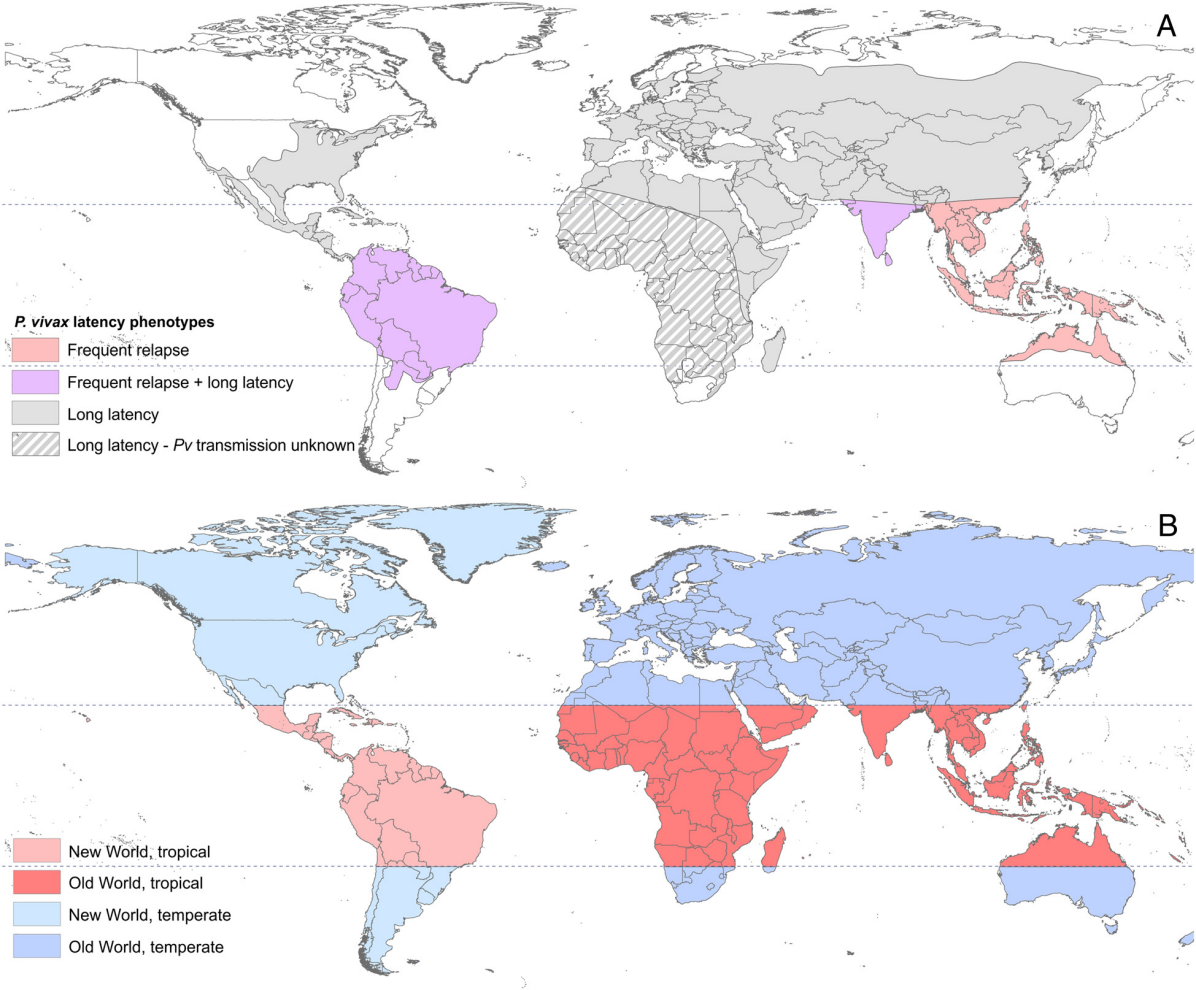
**Proposed distributions of *Plasmodium vivax *relapse latency phenotypes.** Panel **A**, adapted from White [47], shows the historical distribution of frequent relapsing and long-latency relapse strains. The geographic limits were modified using a historical malaria endemicity map from Lysenko and Shemashko [50]. Tropical frequent relapsing strains are in pink and long-latency strains in grey. Much of Africa is shown with grey hatching because the influence of Duffy negativity and its effect on vivax transmission in this part of the world is not yet understood. Purple areas are thought to have both long-latency and frequent relapsing strains. Panel **B** shows the Old and New World classification system based on the analysis and findings from Lover and Coker [51]. Tropical zones (red and pink) harbour strains that relapse more quickly than those in temperate zones (light and dark blue). New World tropical strains (pink) relapse more slowly than Old World tropical strains (red) and Old World temperate strains (dark blue) relapse slower than New World temperate strains (light blue). The dotted lines indicate the ±23.5° latitude lines to delineate temperate and tropical areas. Old World refers to Africa, Eurasia and the Pacific and New World to the Americas and Caribbean regions.

Geographic zones have been proposed for distinguishing areas with similar timing and frequency of *P. vivax* relapse following sporozoite inoculation. Figure 2A, adapted from White [47], and modified by the boundaries of the malaria endemicity map proposed by Lysenko and Semashko (for the maximum range of malaria *circa* 1900) [50], illustrates a proposed distribution of relapse phenotypes. White noted the historical perception that strains that relapse quickly originate from Southeast Asia. Temperate and subtropical areas are characterized by long-latency strains. The Indian subcontinent and South America are thought to contain both long-latency and frequent relapsing strains. A second geographic description of variation in time to relapse is described in a recent study by Lover and Coker [51]. The authors analysed the time to relapse in experimentally infected individuals relative to the geographic origin of the strain. They found that, overall, temperate strains relapse more slowly than tropical ones. However, they also found that New World tropical strains relapse more slowly than Old World tropical strains, and Eurasian temperate strains relapse more slowly than temperate strains from the Western Hemisphere [51]. Figure 2B illustrates this implied classification system.

**Figure 3:**
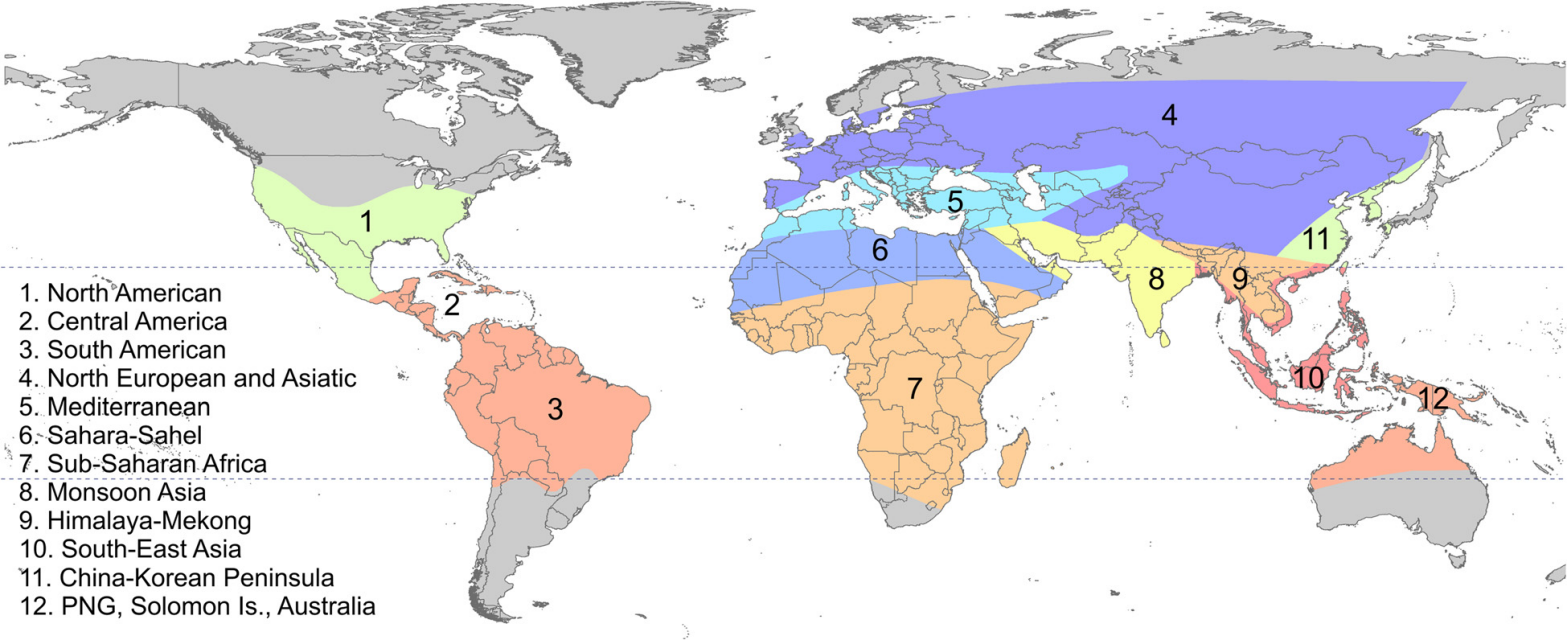
**Zoogeographical zones of malaria transmission.** Zones defined by Macdonald (1957) based on regions with similar ecological and epidemiological characteristics [52].

While the maps in Figure 2 were derived from observations of relapse phenotype, others have grouped regions based on similar ecological and epidemiological characteristics in the absence of relapse observations. These regions may reveal patterns of relapse and help elucidate determinants of the periodicity observed. The zones proposed by Macdonald [52] are shown in Figure 3. They are described by Macdonald as “zoogeographical” malaria regions and share commonalities with many historical biogeographical zonations [53-55]. The approximate boundaries of the zones are delineated by climatic variables that influence malaria transmission rates, such as temperature and rainfall, the intensity of transmission observed, as well as the abundance and behaviours of the locally dominant vector species [52].

A systematic review of *P. vivax* relapse events was conducted with the aim of revealing systematic geographical patterns of relapse frequency and a quantitative description of the potential time to relapse in different regions of the *P. vivax*-endemic world.

## Methods

### Data assembly

A formal literature search for data was conducted on PubMed [56] with the keywords: “vivax AND relapse” on 15 November, 2012 and updated on 24 October, 2013. The search returned 449 references. This list was augmented with the reports of *P. vivax* relapse cited by Baird and Hoffman [32] and from the reference list of two recent reviews of variation in relapse periodicity [47,51]. Additional studies were obtained by correspondence with colleagues active in this research area. Malaria in the military was also examined using references from the US Army and medical records from British soldiers who contracted malaria during duty in World War II. Medical records were obtained from contacts and the Malaria Research Library, now the Malaria Reference Library, kept at the London School of Hygiene and Tropical Medicine. The aim was to obtain as much data as possible regarding *P. vivax* recurrence in all regions where vivax malaria is or has been endemic. The literature sources ostensibly refer to the recurrence events as relapses; however treatment trials and studies conducted in endemic areas may include recrudescences and reinfections in measures of relapse. Because much of the data on *P. vivax* relapse in patients not treated with primaquine originated before use of the drug became common following World War II [57], no restrictions were applied on study dates.

The exclusion criteria for the studies were minimal. Data were not used from patients who had been treated with a sufficient dose (15 mg per kg for 7 or 14 days) of primaquine, or any 8-aminoquinoline drug (pamaquine, plasmochin or pentaquine), due to the effect of the drug on hypnozoites, and therefore patterns of relapse. For example, a series of clinical trials demonstrated that 8-aminoquinoline drug failures (relapses) occurred 60–90 days post-patency, whereas untreated relapses almost always occurred between day 17 and day 35 post-patency [58]. Studies that had treated patients with a five-day course of 15 mg base of primaquine or less, which was shown to be ineffective in preventing relapse [59], were permitted. Relapse in patients treated with a seven-day course of primaquine in South America, where the treatment schedule has been shown to be inadequate [60], were also considered. Blood-stage treatments were not exclusion criteria, but were noted for analysis purposes. Mefloquine prophylaxis and treatments such as mepacrine (quinacrine, atabrine) or chloroquine may cause a delay in relapse because the drugs retain suppressive levels in the patient for long periods after treatment [61,62], making it difficult to distinguish the observed relapse as a first or second relapse. A 14-day cut-off was applied to data abstracted from drug treatment trials. Any re-appearance of infection before the 14th day was categorized as a treatment failure, while infection after day 14 was listed as a relapse. This conservative cut-off was applied to maximize sensitivity. A primary relapse is unlikely to occur before two weeks, even in fast relapsing strains [63]. However, a 14-day cut-off may result in some recrudescence events being classed as a relapse.

When possible, data on time to recurrence from the start of treatment of the primary infection were abstracted to the individual level. Start of treatment was almost invariably the first day of patency, and we considered it most probable that the vast majority of recurrences represented relapses. The majority of studies reported by the day, but those that reported the week or month of relapse were also included (with the last day of the month or week given as the time to relapse). Studies that aggregated months together were excluded. The period of follow-up was recorded for all individuals in each study, including those that did not experience a relapse. Data on the type of patient (prison volunteer, malaria therapy patients, soldiers, etc.) were also collected as it is likely that this would have influenced the time to first relapse. Data on duration of prepatent period from studies performed in experimental settings were recorded where this information was available.

### Georeferencing

Geopositioning of relapse studies was implemented using established methods [64] for those references that did not provide specific coordinates for the study site. The latitude and longitude of entries that provided cities or towns were located as points (<10 sq km) using Google Maps [65]. The centroid of small and large polygons (>25 to <100 sq km and >100 sq km, respectively), such as islands, regions or countries were obtained using ArcMAP 10.0 [66] to determine the latitude and longitude of those areas. The latitude and longitude values recorded corresponded to the origin of infection. Therefore, infections in returning travellers were geopositioned to the place of travel and malaria therapy or experimental trials were positioned to the origin of the strain used.

### Statistical analysis

The number of cases, total person time observed, and mean and standard deviation of time to first relapse were calculated among individuals who experienced at least one relapse in each study. The incidence rate of relapse was calculated from the number of relapse events and total person time. The factors affecting this rate were modelled using mixed-effects meta-analysis in the package metafor [67,68] within the statistical programming language R [69]. As data must be normalized for use in metafor, how to best accomplish this was tested by applying different transformations to the data from each study and assessing deviation from normality by the Shapiro-Wilk test.

The geographic zones described above (Figures 2 and 3) were included in the meta-analysis of relapse rate as categorical moderators. These included the three phenotypic zones shown in White (hereafter referred to as the White-3 system), which were also shown differentiated by Old World and New World (White-5), the four zones described by Lover and Coker, and lastly the 12 zoogeographical regions as described by Macdonald [47,51,52]. Model choice was performed between these geographic systems using information criteria given by metafor (see Table 2). For the best geographic system obtained from this, a meta-analysis of mean time to first relapse among patients with observed relapse events was performed. See Additional file 1 for more information regarding model choice and the meta-analysis. Kaplan-Meier survival curves were generated from pooled individual data from each zone. These curves are intended to illustrate the observed qualitative patterns of relapse in each zone.

**Table 2:** Comparison of geographic classification systems

**System**	**τ**^**2**^	**I**^**2**^	**H**^**2**^	**R**^**2**^	**AIC**	**AICc**
White-3	1.3	97.1	34.0	33.6%	707.7	707.9
Lover	1.6	97.5	40.8	19.4%	728.4	729.0
White-5	0.9	95.4	21.8	57.3%	623.6	624.0
Macdonald	0.8	95.0	20.0	59.9%	612.7	614.1
Modified Macdonald	0.8	95.0	19.9	60.1%	612.9	614.0

Mixed-effects meta-analysis has been performed for 214 different studies or study arms by using the R package metafor [68]. The statistics are: τ^2^, amount of residual heterogeneity; I^2^, residual heterogeneity/unaccounted variability; H^2^, unaccounted variability/sampling variability; R^2^, calculated from the residual heterogeneity (τ^2^) and the residual heterogeneity of an empty model as suggested by Raudenbush [70]; AIC, Akaike information criterion; and AICc, corrected AIC. The values of AIC, BIC and AICc are based on the restricted maximum likelihood, as this corresponds to the REML (restricted maximum likelihood) estimator recommended by experts [67].

### Relapse maps

To visualize geographic variation in relapse, maps were generated by plotting points of median time to relapse in individuals who experienced a relapse from each study included in the final dataset. Regional maps were produced to illustrate the relapse incidence and mean time to relapse modelled within the geographic system chosen in the meta-analysis.

## Results

### Data assembly

**Figure 4:**
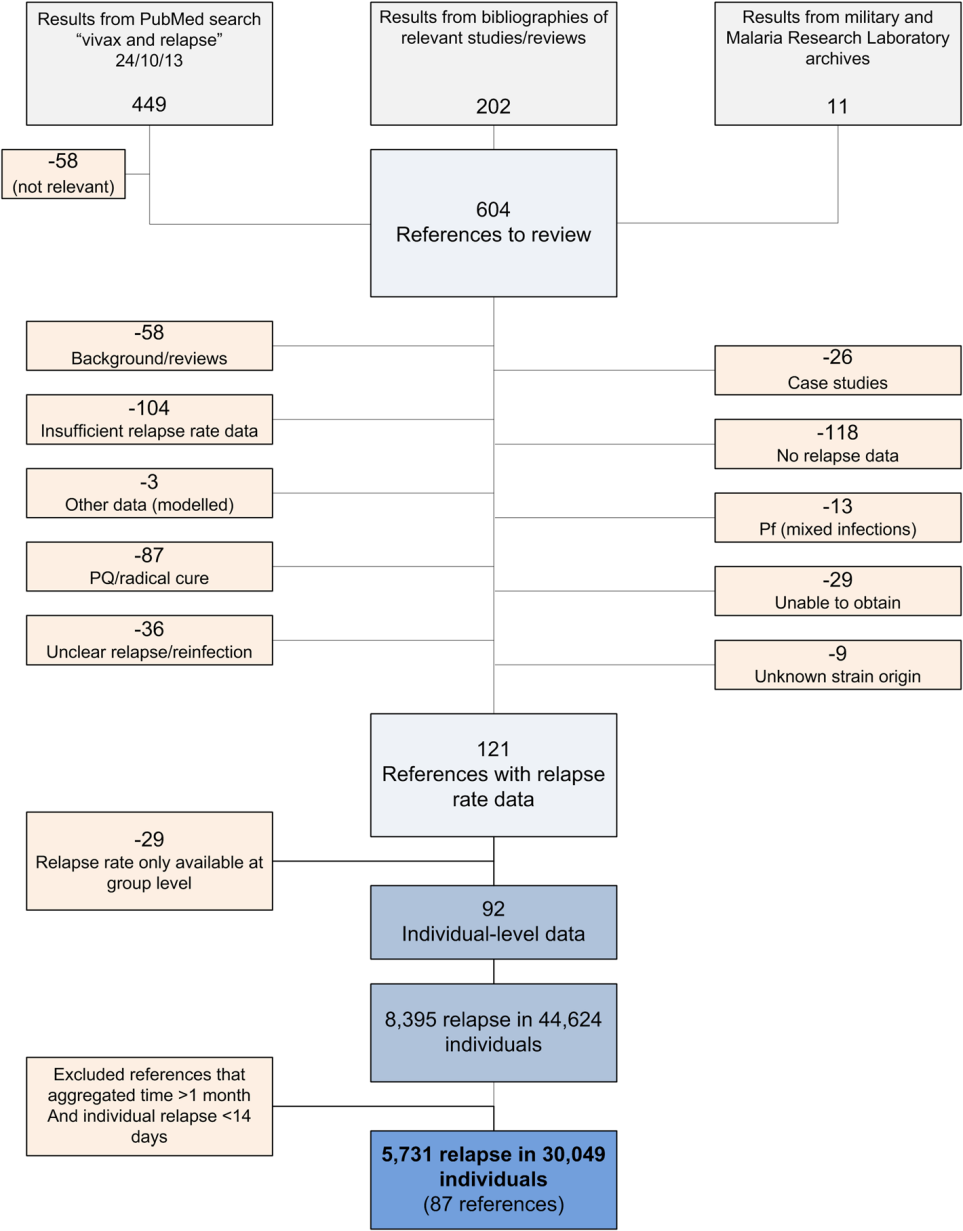
Schematic overview of the literature review procedure and results to obtain individual records of relapse and follow-up.

Following the literature search and collection of unpublished sources, 121 references were found to contain data on time to first recurrence in patients not treated with a sufficient radical cure. Further details regarding the results of the literature review are found in Additional file 1. Details and summary statistics for the 121 references showing time to first recurrence are shown in Additional file 2. Of those, 87 references reported data at the individual level with time to relapse reported in days or values less than or equal to one month (Figure 4). The resulting dataset contained information on 30,049 individuals, of whom 5,731 experienced at least one recurrence. These data are provided in Additional file 3. The observed recurrences are most likely to be relapses, but, in probably rare instances, recrudescences may also be represented among data classified as early relapse (<60 days). The list of references included in the final database is available in Additional file 4.

Relapse rate data were available from 29 different countries and regions. The vast majority of the data were from India (78%, 23537/30049); although of the patients to experience a relapse, only one third (34%, 1931/5731) originated from the subcontinent (Additional file 1). There were data from 23 known strains in experimental infections, but the majority of individuals contracted wild *P. vivax* (94%, 28149/30049). Many of the subjects were not residents of endemic areas. For example, over one third of the patients to relapse (37%, 2731/5731) were military personnel deployed from non-endemic regions. A summary of key aspects of the dataset, such as treatment and patient type, is available in Additional file 1.

### Statistical analysis

**Figure 5:**
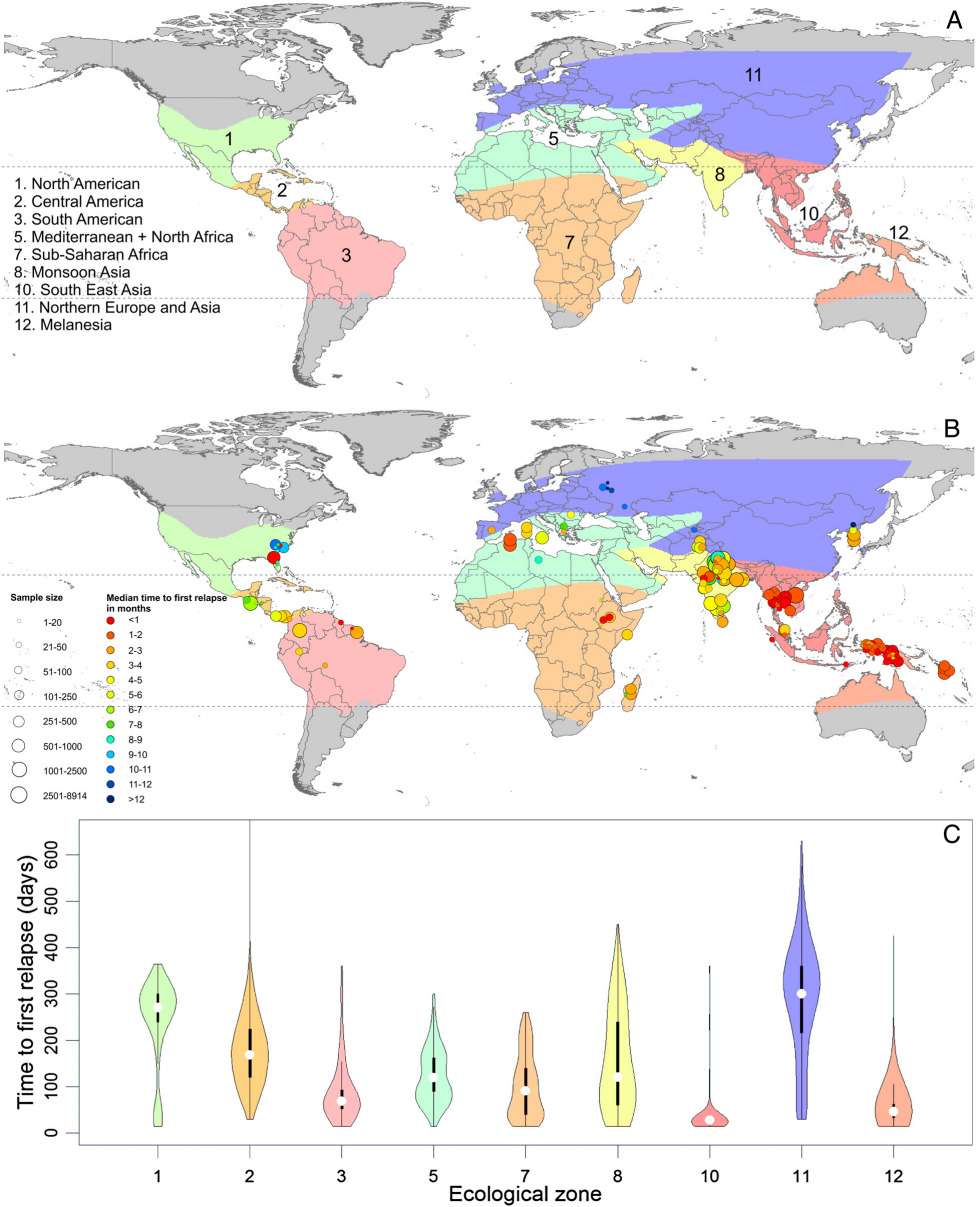
**Revised zoogeographical zones and observed time to first relapse.** Panel **A** illustrates the revised zoogeographical zones used to describe the time to first relapse. Panel **B** shows the median observed time to relapse in each study used to obtain individual data. The size of each point varies by sample size and the time to first relapse is shown on a spectrum of red (less than one month) to dark blue (>12 months). Violin plots in Panel **C** show the observed time to first relapse in individuals from each zone in Panels **A** and **B**. The coloured areas correspond to each zone and to a smoothed approximation of the frequency distribution (a kernel density plot) of the time relapse within each geographic region. The black central bar represents the interquartile range and the white circles indicate the median values. Note that the maximum value for zone 2 extends beyond the plot.

Regardless of the transformation applied to the raw data for each study (identity, log, square root or Freeman-Tukey [71]), the time to first relapse deviated significantly from normal (plots shown in Additional file 1). The deviation was smallest under the log-transformation, which is common for incidence-rate data [72]. The model choice analysis was therefore carried out using log-transformed data. The Macdonald classification system yielded the best description of the data, judging by pseudo-R^2^, the Akaike information criterion (AIC) and corrected AIC (AICc, which accounts for small sample sizes, see Table 2). Possible ways to simplify the Macdonald system such that geographically contiguous zones with similar transmission suitability would be combined were assessed. Further details are provided in Additional file 1. Combining zones 4 and 11 slightly improved the model fit. Zones 5 and 6, and 9 and 10, respectively, were also combined as there were no relapse data available from zones 6 and 9. The revised zones are shown in Figure 5A.

Table 3 presents two estimates of relapse incidence rate for the modified Macdonald system as the number of first relapses per 100,000 person days. One is the crude estimate based on raw data, and the other is the result obtained from the meta-analysis. The highest observed and predicted incidence values are found in zones 9 + 10 and 12, corresponding to Southeast Asia and Papua New Guinea (PNG) plus the Solomon Islands (Melanesia). It is predicted that there will be approximately 800–1,200 relapses per 100,000 person days in this part of the world, compared with the estimated 130 relapses in northern Asia and Europe (zones 4 and 11). The crude and predicted estimates are very different for some zones (namely 3 and 8). This is because the mixed-effect meta-analysis attributes unusually low and high case numbers to inter-study variation and these do not contribute substantively to the rate estimate. A relevant incidence measure could not be calculated for zone 8 (India) because the observed data included several large studies (>2,000 patients) in which the majority of patients did not experience a relapse (Additional file 1).

**Table 3:** Relapse incidence rates for the modified Macdonald system

**Ecological zone**	**Based on raw data, ML with 95% CI**	**Model-based, REML with 95% CI**
1	357 (CI: 318–400)	455 (CI: 313–662)
2	217 (CI: 198–238)	259 (CI: 120–557)
3	419 (CI: 328–528)	1093 (CI: 535–2,233)
5 + 6	214 (CI: 186–245)	262 (CI: 82–839)
7	221 (CI: 191–255)	213 (CI: 95–477)
8	25 (CI: 24–26)	62 (CI: 33–116)
9 + 10	975 (CI: 811–1163)	836 (CI: 351–1,995)
11 + 4	138 (CI: 120–159)	134 (CI: 64–278)
12	1023 (CI: 981–1,067)	1224 (CI: 689–2,174)

The numbers are presented as first relapses per 100,000 person days. The estimate based on raw data is obtained by dividing the number of relapses by follow-up time and using Ulm’s exact formula [73] for confidence intervals. The model-based estimates are calculated from the results obtained from meta-analysis performed by the R package metafor. ML refers to the maximum likelihood and REML to restricted maximum likelihood.

Table 4 shows the raw and modelled estimates of mean relapse time for each of the geographic zones in the modified Macdonald system. These were obtained by running a meta-analysis for mean time to relapse and its standard deviation within each study (in a separate analysis from the original run for the incidence rates), and these are calculated from only the observed relapse events. This produces a modelled estimate of relapse time that is based on the least variable data sources. The Indian zone figures are therefore more plausible in these results. The modelled results show the fastest times to relapse are found in Southeast Asia (zone 9 + 10) and Melanesia (zone 12), around 45 days. South America (zone 3) also had a rapid time to relapse (65 days). However, there were relatively few records from zone 3 (Figure 5B) and some of the heterogeneity in relapse patterns may have been missed. Zone 1, North America, is predicted to have relapse times of about six months. Central America, zone 2, is estimated to have a relapse time of five to six months, driven by a few studies with long relapse intervals observed in Mexico [74]. The Mediterranean zone (5 + 6), a region of seasonal transmission, has a modelled mean time to relapse of five months. Based on the raw data, the mean time to relapse from the few data points in the sub-Saharan Africa zone (7) was only one month. Finally, the northern Europe and Asia zone (11 + 4) has by far the longest modelled mean time to relapse of ten months.

**Table 4:** Mean time to relapse among geographic zones

**Ecological zone**	**Based on raw data, ML with 95% CI**	**Model-based, REML with 95% CI**
1	100 (CI: 99–101)	185 (CI: 162–208)
2	239 (CI: 239–240)	164 (CI: 117–212)
3	53 (CI: 53–53)	65 (CI: 18–113)
5 + 6	153 (CI: 153–153)	151 (CI: 80–221)
7	31 (CI: 30–31)	107 (CI: 57–158)
8	181 (CI: 180–181)	120 (CI: 82–159)
9 + 10	289 (CI: 288–290)	41 (CI: -11–92)
11 + 4	89 (CI: 87–90)	299 (CI: 254–345)
12	122 (CI: 121–123)	47 (CI: 12–81)

The model-based estimates have been calculated by using the R package metafor, which acknowledges interstudy variation. Thus the numbers differ from raw means calculated from the data. ML refers to the maximum likelihood and REML to restricted maximum likelihood. Please note that sample means by the very definition concern only observed events, and consequently this table ignores person time from censored observations.

**Figure 6:**
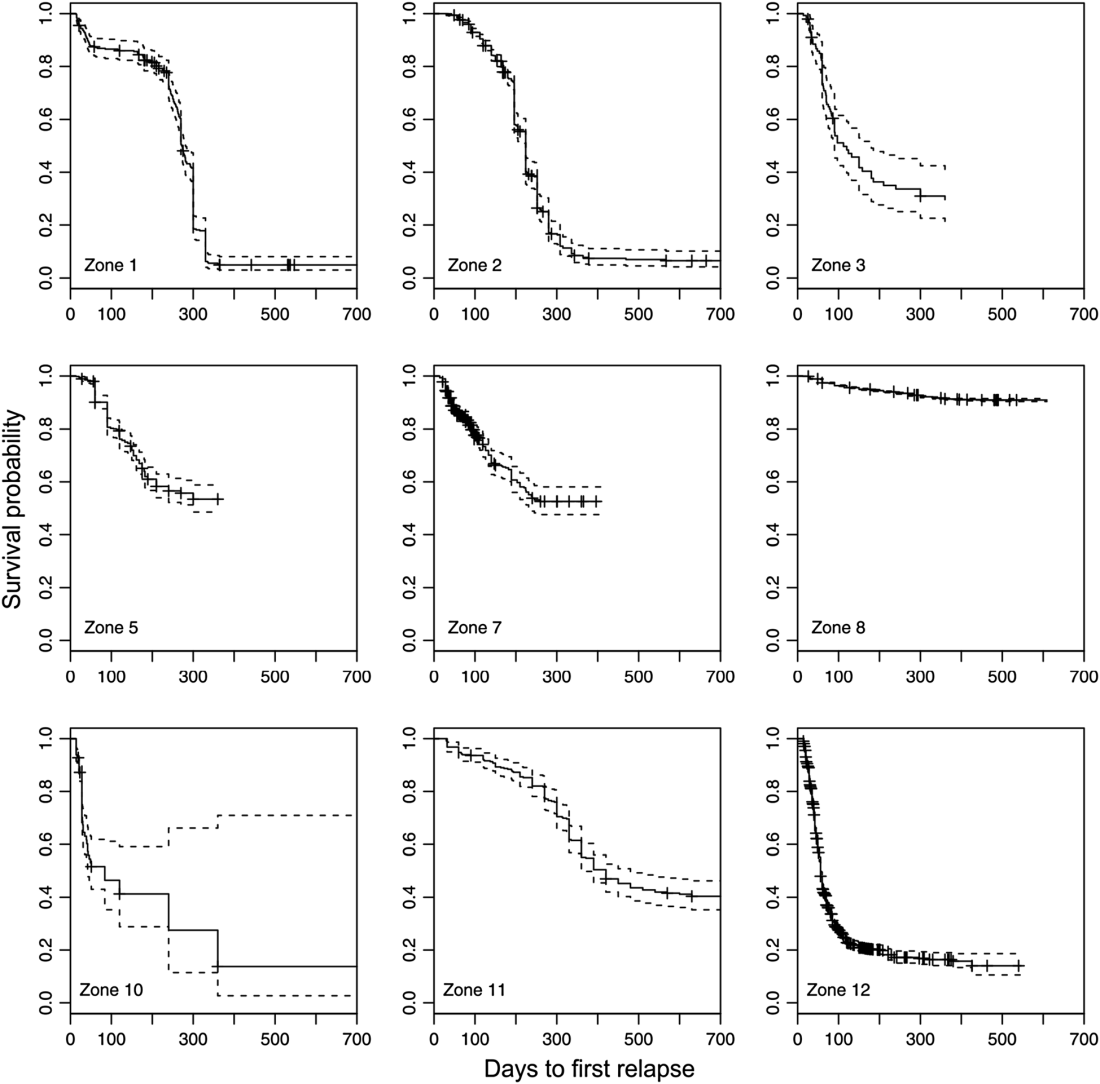
**Survival curves for the modified Macdonald system.** Shown are the Kaplan-Meier estimates (solid lines) with 95% confidence intervals (dotted lines). For each curve, all individual-level data from the respective zone have been pooled. The curves terminate at the longest follow-up day in each zone. The ticks indicate censoring events, i.e., losses to follow-up or the completion of a study without relapse. Zone 1: North America, zone 2: Central America, zone 3: South America, zone 5: Mediterranean and North Africa, zone 7: sub-Saharan Africa, zone 8: Monsoon Asia, zone 10: Southeast Asia, zone 11: northern Europe and Asia, zone 12: Melanesia.

Finally, Figure 6 presents the survival curves for the modified Macdonald system. Note that the meta-analysis models described above do not yield survival curves. The curves in Figure 6 are based on the Kaplan-Meier survival function estimator, and they are calculated from pooled raw data within the geographic zones to provide a quantitative comparison of relapse patterns among zones. For example, in zone 1, the majority of the patients observed had relapsed by day 300, whereas in zone 12, most patients had relapsed before day 100. The curve in zone 8 does not reveal much regarding the time to relapse because of the small number of relapsing patients.

### Relapse maps

**Figure 7:**
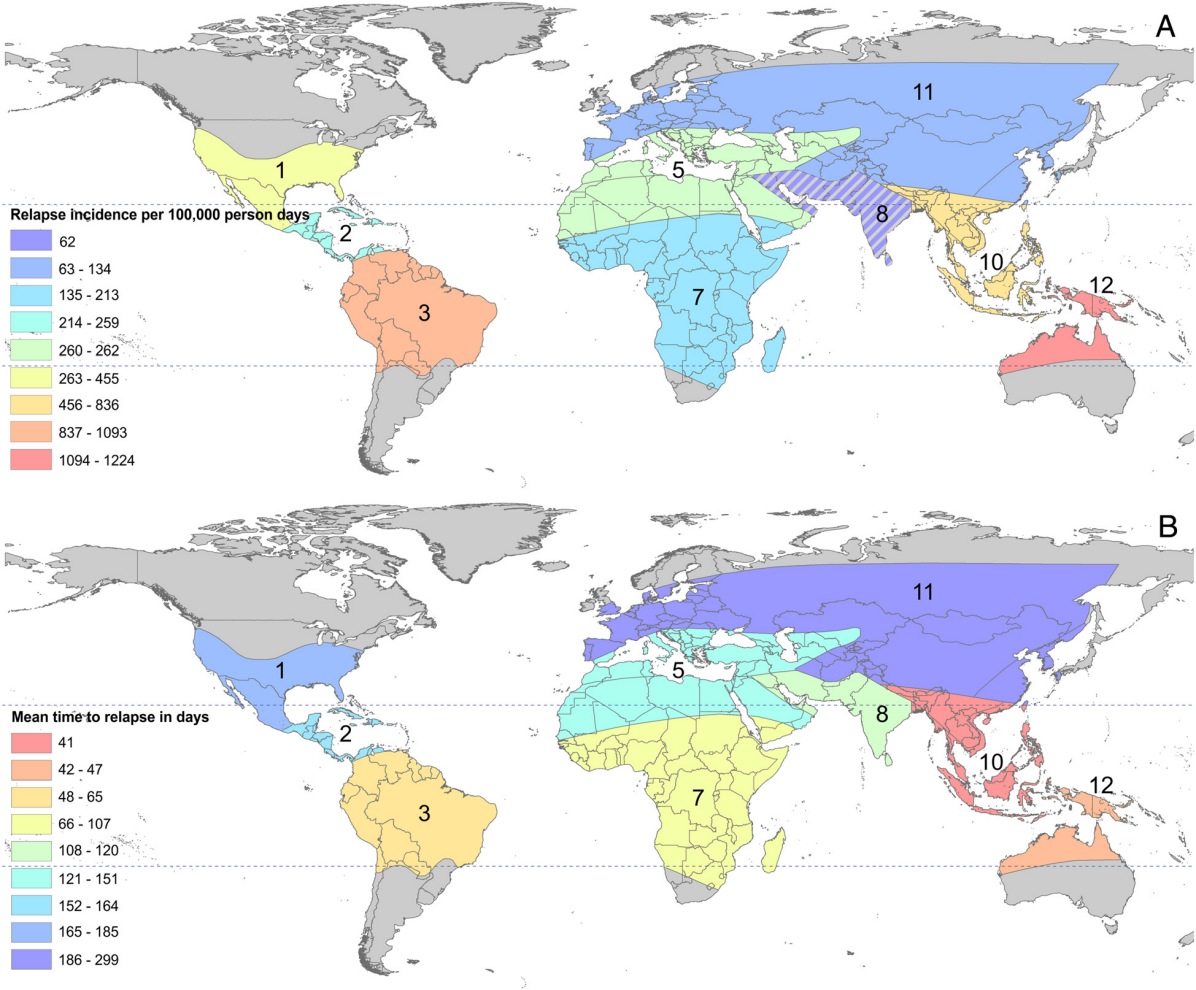
**Modelled relapse incidence and mean time to relapse.** Panel **A** illustrates the relapse incidence per 100,000 person days on a spectrum of blue to red, with red being the highest incidence of relapse. Zone 8 is hatched to indicate that the predication is particularly uncertain. Panel **B** illustrates the predicted mean time to relapse on a spectrum from blue to red, with red being most frequent relapse. The numbers of the zones correspond to those shown in Figure 5A.

The revised zoogeographical zones used in the analyses described above are shown in Figure 5A. In Figure 5B the median time to relapse for study locations is specified with points inside the geographic zones. The map illustrates a concentration of fast-relapsing strains in Southeast Asia and Melanesia. The heterogeneity in relapse periodicity observed in zone 8 is also apparent in this map. The variation in the North American zone is due to the behaviour of strains occasionally relapsing quickly after a long incubation period between inoculation and primary attack (data available from experimental inoculations only), but for the most part relapses followed a long period of latency after a short incubation period. Summary statistics of the time to relapse by zone are shown in Additional file 1. Panel C in Figure 5 is a violin plot of observed time to relapse in each zone. This illustrates that those shorter relapses in North America are fairly rare. The violin plots also show that the longer periods to relapse in Central America and Southeast Asia are rare. Heterogeneity in other zones, such as 8 and 11 + 4, is also demonstrated by the violin plots. Figure 7 illustrates the results of the predicted relapse incidence and mean time to relapse by region. In Figure 7A, zone 8 is hatched out as the resulting estimate is biologically implausible and is most likely caused by the handful of studies with large numbers of patients not reporting relapse during the observation period.

## Discussion

The aims of this paper were to review the timing and frequency of *P. vivax* relapse of known origin in patients not treated with a hypnozoitocide to characterize variance in these patterns within geographic dimension, to identify a system to classify the variation in relapse observed and to describe the pattern of relapse in each area. A modified classification of the zoogeographical zones of malaria transmission outlined by Macdonald [52] was found to best describe the observed variation in relapse incidence. The rate of relapse and mean time from primary infection to relapse was predicted in each of the nine zones. These quantitative estimates of the contribution of relapses to *P. vivax* case incidence are crucial in informing estimates of disease burden and the origin of acute attack, i.e., from biting mosquitoes or emergent hypnozoites. This understanding, in turn, informs critical decision-making in control strategies that effectively weigh the benefit of anti-mosquito *versus* hypnozoitocidal interventions. They also help identify regions in which strains have long-term latency and are therefore undetectable to standard diagnostic methods (rapid diagnostic tests and microscopy) for long periods of time.

The results presented here further refine historical interpretations and recent analyses of the geographic variation in relapse periodicity. As shown in Figure 2, tropical strains relapse more rapidly than temperate strains and New World strains vary from those in the Old World. White’s illustration of the variation in relapse phenotype shown in Figure 2A shows that Southeast Asia and Asia-Pacific is the only region having exclusively frequent relapse behaviour. The results also showed infections from this region to relapse quickly, with a few rare exceptions. White showed that both frequent relapsing and long-latency strains are present in India and South America. The data from India appear to affirm this, with relapse patterns from the subcontinent and surrounding areas so heterogeneous that it was impossible to generate logical model predictions of relapse incidence for the region. The low incidence in region 8 shown in Table 3 is not believed to be a reflection of the presence of long relapsing strains, but rather a result of natural infections that either did not result in a relapse or the resulting parasitaemia was too low to be detected by the study. There is not presently an explanation for the lack of relapses, but this phenomenon has also been observed in recent tafenoquine trials in India [75]. The variation in relapse timing in the raw data observed in India (Figure 5B) is likely a result of the wide variation in transmission settings found within this zone. There are tropical forest areas, similar to zone 12, dry habitats like those in zone 5 and highland areas that border zone 11. In addition, the presence of *Anopheles stephensi*, adapted to breeding in artificial water collections [76,77], has extended transmission into urban areas. Therefore, in addition to issues of data availability and study design, the range of ecological settings in the zone, and likely some of the other zones, may also contribute to the variance in relapse behaviours observed.

The results presented for South America were different from what was shown in White’s phenotype map (Figure 2A). South America was predicted to have a high relapse incidence, comparable to Southeast Asia, and a two-month mean time from primary attack to relapse. The data available from this region were limited and determining the cause for the observed difference is therefore difficult. There has been renewed interest in the origin of the American strains of *P. vivax*, whether they originate from somewhere in Asia or were sent west from Africa by migration and the slave trade, as has been proposed for *P. falciparum*[78]. This could influence the nature of the relapse periodicity observed. Improved understanding of the phylogeny of *P. vivax* may reveal information about the pattern of relapse in this region.

The analysis by Lover and Coker [51] revealed that tropical regions relapse more quickly than temperate strains. However, Central America and sub-Saharan Africa had relapse patterns similar to the Mediterranean with moderate relapse incidence (around 250 relapses per 100,000 person days) and five to six months between primary infection and relapse. These regions seem to be better described as an “intermediate” relapsing phenotype between the frequent relapsing strains in Southeast Asia and South America and the long-latency temperate strains in North America and Europe/Asia.

The results of the northern temperate regions concur with the findings of Lover and Coker [51]. The authors noted that while in general, temperate strains relapse more slowly than tropical strains and that New World tropical strains were slower than Old World strains, the opposite was true of the temperate strains. Based on this analysis, the New World temperate strains relapsed more rapidly than the Old World temperate strains (Figure 7). The modelled results showed that the relapse incidence was 455 per 100,000 person days and mean time to relapse was six months in North America. However, again, this high incidence compared to that in northern Asia and Europe (134 relapses per 100,000 person days) could be due to a few exceptional experimental subjects who received large sporozoite inoculations [79-81].

The utility of the predictions made is limited by the nature of the data available. There are few contemporary data on *P. vivax* infections in patients not treated with a hypnozoitocide. Therefore, much of the data used were from drug trials on adult workers, military personnel and prison “volunteers”, as well as data from when malaria was used to treat neurosyphilis patients. The age and immunity of the patients would perhaps not be representative of relapse as it would occur among residents of the strain region of origin. This could be important because the children in many endemic settings likely carry the greatest burden of relapsing infections [82]. Some experimental challenge subjects were inoculated with relatively heavy sporozoite dosages, a factor that greatly influences the time from primary attack to relapse [47] and is likely to differ from relapses following more modest numbers of sporozoites acquired from wild anophelines. It is straightforward to attribute an infection as a relapse or re-infection in experimental settings, but this could not be distinguished for *P. vivax* infections acquired in the wild. Effort was made to obtain studies where the follow-up period was conducted in a non-endemic area (for example, in a hospital in a city). Of the 5731 records of relapse 88% (n = 5030) were obtained from experimental or non-local populations (such as military personnel). Lastly, the strains used in therapy and drug trials in the first half of the twentieth century were of “known” origin and infections were geopositioned to those sites. However, it is not certain if the strains used were of their named origin. For example, the “Madagascar” strain was obtained from an Indian seaman whose last port of call was in Madagascar [83] and may conceivably, therefore, originate from elsewhere.

A principal limitation of this work is the inability to conclusively identify a recurrent infection as a relapse, recrudesence or reinfection. This is particularly an issue given the rising rate of resistance to standard treatments such as chloroquine [84]. In cases of chloroquine failure, recurrence can occur one to two months after initial treatment [63], making differentiation between relapse and recrudescence a challenge. Chloroquine and chloroquine-combination therapies were by far the most common treatment regimens (82%, 24787/30049). Of those patients to receive chloroquine, 2519 patients experienced a relapse or recurrence, 523 of which occurred before 60 days. This is equivalent to 9% (523/5731) of the relapse records. In addition, the effect of resistance on recurrence is likely abated by the historical nature of the dataset. The first cases of chloroquine-resistant *P. vivax* were reported in 1989 [85]. Of the 5731 records of relapse, 2080 occurred before 60 days and 82% of those (n = 1701) were observed before 1989. Therefore, increased resistance is unlikely to have a large effect on the results and instances of recrudesence being classed as a relapse would have been rare. While the relapse signal represented in these data certainly contains some noise due to reinfection or recrudescence, we considered these other sources of recurrences improbable relative to relapse. Figures 5C and 6 seem consistent with this assumption because recurrence due to reinfection or recrudescence would have been far more stochastic than relapse, obscuring or effacing the patterns shown by the randomness of timing of those events relative to primary parasitaemia.

There are aspects of the data that were not incorporated into the analysis performed here that could be addressed in future work. First, it was difficult to account for strains with long incubation periods before primary infection (information only available for a subset of data from experimental settings) followed by relatively short time to relapse. This was occasionally exhibited by the North American St Elizabeth strain and *P. vivax multinucleatum* from China (see Additional file 3). The link between the sporozoite dose and latency, mentioned above, was shown in the literature to be an important factor in determining relapse patterns [46,86,87], but was not incorporated into the analysis as it cannot be known for wild infections. This may be a possible explanation why the incidence of relapse in North America was greater than that predicted for Central America. However, this was likely not a common problem with the experimental studies used. The majority of studies aimed to induce patency and the sporozoite inoculations were large, but not extreme. There were only a handful of studies included that used particularly large inoculations in order to study the effect of dosage on relapse pattern [79-81].

The type of patient varied among studies (prison volunteers, military personnel, malaria therapy patients, outpatients in an endemic area, etc.) and the drug type and dosage varied within and among studies. In some studies, primary attacks were treated with insufficient doses of 8-aminoquinolines or drugs that have long half-lives. Mepacrine has a half-life of up to a month and can delay relapses by about 30 days [88,89] and chloroquine, the most common drug in the dataset, can delay parasite re-appearance by anywhere from two to six weeks [47]. Inclusion of the subject-type and treatment as explanatory variables was tested; however, the results were similar to the simpler model used (see Additional file 1). Finally, the analysis only addressed the periodicity between primary attack and first observed recurrence (relapse). The modelled estimates of incidence do not account for multiple relapses. Both the frequency and number of relapses will vary based on a variety of factors including inoculums, age of patient and origin of infection. While many studies did not follow patients long enough to report multiple relapses, further work in this area will be essential to obtain measures of the *P. vivax* force of infection.

In addition to the limitations posed by the data and survey study designs, the analysis is limited by the types of statistical methods available for this kind of task (see Table 5). It would be preferable to use a statistical model that is both hierarchical (to account for between-study variation) and employs a suitable survival-analysis likelihood. Unfortunately, software used to fit this type of model was numerically unstable and hence the mixed-effects meta-analysis was employed.

**Table 5:** Strategies for modelling survival data obtained from many dissimilar sources

**Statistical method**	**Accounts for individual-level variation**	**Accounts for between-study variation**	**R packages**
Fixed-effects meta-analysis	No; operates on summary statistics	No	Many software packages, e.g., meta and metafor
Mixed-effects meta-analysis	No; operates on summary statistics	Yes	Many packages, e.g., meta and metafor; also general-purpose software such as lme4 may be used
Survival analysis for pooled data	Yes	No	A number of packages, e.g., survival, eha and flexsurv
Survival analysis with mixed effects	Yes	Yes	Most notably R/coxme; flexible software seems to be hard to find

Data were analysed using mixed-effects meta-analysis, which is common for this type of study. All of the methods have strengths and weaknesses.

The mechanism of hypnozoite activation to cause an acute attack (relapse) remains unknown. There is clearly variation in relapse “phenotype”. Based on the results presented here, timing of relapse appears to vary geographically in conjunction with areas of similar ecology and malaria transmission patterns. However, it is difficult to determine whether long latency occurs in regions of frequent relapse (tropical areas such as zones 9 + 10 and 12). A long-latency relapse may be thought to be another short-term relapse in a succession of rapid relapses, and the genotype cannot reveal if it is in fact a separate relapse “event” [47]. Nonetheless, understanding broad patterns of relapse is of use epidemiologically. There tends to be fewer overall relapses in the long-latency strains because hepatocytes that host the hypnozoites may die before the relapse event occurs. The resulting burden of hypnozoites from different strains or regions has implications for sensitivity to primaquine and therefore the dosage of primaquine that should be used [47]. This was observed in treatment of soldiers returning from Korea (fewer hypnozoites) [90] relative to those returning from the Pacific (high hypnozoite burden) [58] and will have implications for future control strategies.

## Conclusions

Frequency of relapse varies geographically. The association between relapse rate and the geographic regions does not clarify causation. Geographic variation does not directly imply environmental cues as triggers for relapse, even if the revised Macdonald system resembles the distribution of transmission suitability. Relapse frequency may result from evolved responses to average transmission season duration or arise from proximate cues, such as triggers from other infections, correlated with *P. vivax* transmission and/or vector suitability periods. There is likely an interaction between activation of latent hypnozoites from infection and an evolved trait for strains from areas of seasonal transmission to remain dormant during periods of low mosquito abundance. Regardless of the cause, these patterns are important for the treatment of individual infections, measures of *P. vivax* burden and the prospects for control and eventual elimination of the disease from endemic areas.

## Abbreviations

95% CI: 95% confidence interval; AIC: Akaike information criterion; AICc: Corrected Akaike information criterion; BIC: Bayesian information criterion; G6PD: Glucose-6-phosphate dehydrogenase; ML: Maximum likelihood; PNG: Papua New Guinea; REML: Restricted maximum likelihood.

## Competing interests

The authors declare that they have no competing interests.

## Authors’ contributions

KEB and SIH conceived the study and oversaw its implementation with assistance from MSK, PWG and JKB. KEB wrote the first draft of the manuscript and assembled data with assistance from REH, JPM and GDS. Environmental data were provided by TPVB. MSK led the design of the modelling framework with input from NG, SB and DLS. All authors participated in the interpretation of results and in the writing and editing of the manuscript. All authors read and approved the final manuscript. KEB and SIH will act as guarantors for the paper.

## Supplementary Material

Additional file 1 Additional results and statistical analyses. Click here for file

Additional file 2: Table S1 Summary relapse dataset. Click here for file

Additional file 3: Table S2 Individual-level relapse dataset used in analysis. Click here for file

Additional file 4 Individual-level data reference list. Click here for file
