# Supplementary material for: Geographical variation in Plasmodium vivax relapse
Source: Malar J. 2014 Apr 15;13:144. doi: 10.1186/1475-2875-13-144 (PMC4021508; doi:10.1186/1475-2875-13-144)
Supplement: Additional file 1 — Additional results and statistical analyses. [file 1475-2875-13-144-S1.doc]

**Additional File 1**

**Geographical variation in *Plasmodium vivax* relapse**

Authors: Katherine E Battle^1§^, Markku S Karhunen^1^, Samir Bhatt^1^, Peter W Gething^1^, Rosalind E Howes^1^, Nick Golding^1^, Thomas P Van Boeckel^2^, Jane P Messina^1^, G Dennis Shanks^3^, David L Smith^4,5^, J Kevin Baird^6.7^, Simon I Hay^1,5§^

^§^Authors for correspondence: katherine.battle@zoo.ox.ac.uk and simon.hay@zoo.ox.ac.uk

This file includes:

Additional results and statistical analyses

**Initial data collection**

Following the literature search, data were first recorded as aggregated records of relapse. In this initial dataset, there were 163 records of relapse from 121 references on 56,649 patients, of which 11,152 experienced at least one relapse. Relapse was measured from a cohort of patients and reported as minimum, mean, median and maximum time to first relapse of the patients that relapsed. However, few of the studies provided exact measures of the total follow-up time. Thus, it was necessary to collect the individual-level records and calculate the person time from these for analysing the incidence rate. Ninety-two of the original references in the aggregated dataset contained individual level data. Following application of exclusion criteria, eliminating records that aggregated time >1 month and instance of relapse earlier than 14 days, the 44,624 patients records (8,395 relapse) was reduced to 30,049 (5,731 relapse) from 87 references as shown in Figure 3 in the main text.

The publication year of studies included in the analysis ranged from 1920 to 2013, with a mean and median value of 1949 and 1962, respectively. However, given that several later studies included a large number of patients, based on individual-level data the mean and median publication years were 1982 and 1990. Figure A1 illustrates the temporal distribution and size of studies included by country.

The number of patients observed and those that relapsed are shown by country, treatment type, patient type and Macdonald zone in Table A1. As shown in Figure A2, the majority of the patients were from India, but a low proportion experienced a relapse. Of the 23 known strains, the most commonly studied was the Chesson strain (39%), followed by the Madagascar (17%) and St. Elizabeth (16%) strains. The wild infections originated from 19 different countries and regions. The most common drug therapy used was chloroquine given in combination with primaquine at dosages deemed insufficient as an effective radical cure (3 or 5 day primaquine courses) . Outpatients and military personnel were the most frequently observed patient type. Malaria therapy patients and prison “volunteers” comprised only 2% and 3% of the dataset, respectively.

Summary statistics of the observed time to relapse as well as details of follow up times for the individual level data are reported by ecological zone in Table A2.

**Table A1 Summary of important aspects of individual data**

The values represent the number of total individuals observed and the number of individuals who experienced at least one relapse.

| **Country/Region** | **total** | **relapse** | **Treatment** | **total** | **relapse** |
| --- | --- | --- | --- | --- | --- |
| Brazil | 60 | 38 | 8-aminoquinolines | 18129 | 1281 |
| Cameroon | 6 | 1 | Chloroquine | 8029 | 1926 |
| China | 3 | 3 | Mepacrine | 1837 | 1330 |
| Comoros | 3 | 1 | Proguanil | 42 | 36 |
| El Salvador | 314 | 306 | Quinine | 680 | 467 |
| Ethiopia | 292 | 16 | SN | 165 | 109 |
| French Guiana | 13 | 3 | None | 307 | 228 |
| Greece | 94 | 72 | Other | 76 | 62 |
| Guyana | 22 | 2 | Unknown | 784 | 292 |
| India | 23537 | 1931 |  |  |  |
| Indonesia | 106 | 33 | **Subjects*** | **total** | **relapse** |
| Iran | 3 | 3 | ACD | 1617 | 177 |
| Korea (North and South) | 101 | 46 | ACD and PCD | 348 | 326 |
| Macedonia | 20 | 14 | Malaria therapy | 596 | 355 |
| Madagascar | 328 | 174 | Military | 3723 | 2137 |
| Mediterranean | 437 | 126 | Outpatients | 22719 | 1953 |
| Mexico | 70 | 44 | Prison volunteers | 816 | 584 |
| Myanmar | 235 | 80 | Travellers | 3 | 1 |
| New Guinea | 1081 | 752 | Unknown | 227 | 198 |
| Nicaragua | 6 | 4 |  |  |  |
| Pacific | 1019 | 753 | **Macdonald zone** | **total** | **relapse** |
| Pakistan | 10 | 10 | 1. North America | 335 | 299 |
| Panama | 750 | 129 | 2. Central America | 1140 | 483 |
| Peru | 51 | 29 | 3. South America | 146 | 72 |
| Russia | 242 | 151 | 4. N. Europe and Asia | 245 | 154 |
| Solomon Islands | 863 | 695 | 5. Mediterranean | 551 | 212 |
| Thailand | 39 | 8 | 6. Sahara-Sahel | 0 | 0 |
| USA | 335 | 299 | 7. Sub-Saharan Africa | 629 | 192 |
| Vietnam | 9 | 8 | 8. Monsoon Asia | 23550 | 1944 |
| TOTAL | 30049 | 5731 | 9. Himalaya-Mekong | 0 | 0 |
|  |  |  | 10. South East Asia | 360 | 124 |
|  |  |  | 11. China-Korean Pen. | 101 | 46 |
|  |  |  | 12. PNG + Solomon Is. | 2992 | 2205 |

*****ACD = active case detection, PCD = passive case detection

**Table A2 Summary statistics of observed time to first relapse by zone**

The total number of patients and relapses observed by zone are shown along with the minimum, 1^st^ quartile (1^st^ Qu), median, mean, 3^rd^ quartile (3^rd^ Qu) and maximum time to relapse in days. The 95% confidence interval (CI) of time to relapse is also shown. The minimum, median, mean and maximum follow up (FU) observed in each zone is also shown.

| **Zone** | **Total patients** | **Total relapses** | **Min** | **1^st^ Qu** | **Median** | **Mean** | **3^rd^ Qu** | **Max** | **95% CI** | **Min FU** | **Med FU** | **Mean FU** | **Max FU** |
| --- | --- | --- | --- | --- | --- | --- | --- | --- | --- | --- | --- | --- | --- |
| 1 | 335 | 299 | 14 | 240 | 270 | 239.4 | 300 | 364 | 228.5, 250.2 | 14 | 360 | 395.6 | 1778 |
| 2 | 1140 | 483 | 30 | 120 | 168 | 180.6 | 224 | 1582 | 171.3, 190.0 | 35 | 180 | 246.6 | 2884 |
| 3 | 146 | 72 | 14 | 51.75 | 68.5 | 88.56 | 92.5 | 360 | 72.7, 104.4 | 28 | 180 | 188.6 | 360 |
| 5 + 6 | 551 | 212 | 14 | 90 | 119 | 122 | 162.8 | 300 | 114.0, 130.0 | 30 | 240 | 213.1 | 365 |
| 7 | 629 | 192 | 14 | 40.25 | 90.5 | 100 | 140 | 260 | 90.3, 109.7 | 28 | 245 | 193.5 | 407 |
| 8 | 23550 | 1944 | 14 | 60 | 120 | 153 | 240 | 450 | 148.4, 157.6 | 30 | 360 | 340.1 | 609 |
| 9+10 | 360 | 124 | 14 | 21 | 28 | 30.81 | 28 | 360 | 24.1, 37.5 | 14 | 28 | 44.45 | 730 |
| 11+4 | 346 | 200 | 30 | 216 | 300 | 289 | 360 | 630 | 271.5, 306.5 | 120 | 730 | 637.7 | 990 |
| 12 | 2992 | 2205 | 14 | 33 | 46 | 52.58 | 62 | 426 | 51.3, 53.9 | 14 | 112 | 116.9 | 546 |

**Additional statistical analyses**

*Alternative modelling strategies*

We attempted to construct individual-based survival models for relapse time. This seemed like a natural choice, given the fact that we had collected individual-based data. Initially, we tried to pool the data across geographic areas, but the log-rank test soon revealed that the inter-study variation could not be ignored in these data (see also Table 4 in the main text). Figure 5 in the main text is based on this type on pooling; while we regard this as an informative figure, the confidence intervals are not justified in the strictest statistical sense. Subsequently, we attempted to model the data by using mixed-effects Cox regression implemented in the R package coxme . However, this led to numerical convergence problems for some model variants (geographic classification systems). Finally, we attempted to replicate the results of R/metafor by using mixed-effects Poisson regression (R/lme4), which operates on average incidence rate within each study, just as metafor . We were unable to make the optimisation algorithm converge using lme4 in some of the model variants. Based on these results, we judged that the only reliable results were obtained from metafor, and consequently, we report these as the main finding of this study.

Throughout most of the various analyses performed, we found that the Macdonald system was the most justified description of the data, judging by statistical criteria. This effect persisted in initial survival analyses done on data pooled by geographic zone (see above), and also in the final meta-analysis. Furthermore, this same relationship seemed to carry over to mixed-effects survival analysis (R/coxme), but as noted above, we do not present the results from those analyses here.

*Sensitivity of the meta-analysis*

We tried to improve the predictive power of the Macdonald system by combining some of the geographic zones, but only in one case did this yield a slight improvement. The various zone combinations and the resulting effects on the predictive power of the model are shown in Table A3. Trial F was the system that was applied in the analysis because it combined zones with little or no data with geographically contiguous zones with data. This slightly improved the predictive power of the system.

In addition to analysing discrete geographic zones, we also tried to model the data by using a measure of the longest transmission suitability period as a continuous moderator. This variable was generated from combining monthly measures of temperature suitability and enhanced vegetation index (EVI; a measure of vegetation and proxy for moisture). The resulting global index indicated the number of months per year each 5 km x 5 km pixel is suitable for malaria transmission (Figure A3). However, the model with the continuous covariate provided statistics that were only modest compared to those of the geographic systems (compare Tables A3 and A4). Thus we conclude that there is an association between the transmission suitability and the relapse rate, but the geographic zones capture this association better, than any of the continuous transformations tried here. In addition, they may capture other relevant features of the *P. vivax* life cycle.

**Table A3 Testing for improved Macdonald systems**

This table presents statistics on meta-analysis models where different modifications of the Macdonald system are used as moderators. The statistics are: pseudo-R^2^, the amount of heterogeneity accounted for; AIC, Akaike information criterion; BIC, Bayesian information criterion; and AICc, corrected AIC. The values of AIC, BIC and AICc are based on the restricted maximum likelihood estimator.

| **Trial** | **Zone combinations** | **Pseudo-R^2^** | **AIC** | **BIC** | **AICc** |
| --- | --- | --- | --- | --- | --- |
| **A** | **Original (no combinations)** | 59.9 % | 612.7 | 649.2 | 614.1 |
| **B** | **2+3** | 56.8 % | 627.2 | 660.4 | 628.3 |
| **C** | **5+6** | 60.1 % | 612.9 | 646.1 | 614.0 |
| **D** | **10+12** | 60.0 % | 613.6 | 646.9 | 614.8 |
| **E** | **4+5+6+11** | 60.0 % | 613.9 | 643.8 | 614.8 |
| **F** | **5+6, 9+10, 4+11** | 59.9 % | 612.7 | 649.2 | 614.1 |
| **G** | **2+3, F** | 56.8 % | 627.2 | 660.4 | 628.3 |
| **H** | **10+12, F** | 56.8 % | 627.2 | 660.4 | 628.3 |
| **I** | **All changes** | 57.0 % | 628.4 | 655.0 | 629.1 |

**Table A4 Using suitability as a continuous covariate**

This table presents statistics on meta-analysis models where different transformations of the climatic suitability of transmission (Figure A3) are used as a continuous moderating variable, in place of the geographic zones (Macdonald system). The statistics are: pseudo-R^2^, the amount of heterogeneity accounted for; AIC, Akaike information criterion; BIC, Bayesian information criterion; and AICc, corrected AIC. The values of AIC, BIC and AICc are based on the restricted maximum likelihood estimator.

|  | **Pseudo-R^2^** | **AIC** | **BIC** | **AICc** |
| --- | --- | --- | --- | --- |
| **Macdonald** | 60 % | 613 | 649 | 614 |
| **Empty model** | 0 % | 778 | 785 | 778 |
| **X** | 0 % | 777 | 787 | 777 |
| **log (X + 1/30)** | 2 % | 772 | 782 | 772 |
| **√X** | 1 % | 774 | 784 | 774 |
| **X^2^** | 0 % | 778 | 788 | 778 |
| **X + X^2^** | 0 % | 778 | 788 | 778 |

We reran the mixed-effects meta-analysis by using different transformations of data. For square-root and Freeman-Tukey transformed data, White’s 5-class system (3 phenotype zones, differentiated by Old and New World) yielded the best results; whereas White’s original 3-class system was the best description of the untransformed data. However, we argue that there is no particular reason to trust these results more than the log-transformed data presented in the main matter, as the Shapiro-Wilk test showed that the log-transformation produced the least deviations from normality (Figure A4). We also tried to include patient medication (if known) or the type of study population as additional moderators in the meta-analysis framework, and calculated the model choice criteria again. The Macdonald system also performed best in these analyses (Table A5).

**Table A5 Results of the addition of subject and medication type to meta-analysis**

The AICc values reflect the addition of adding the type of subject, medication used or both to the various geographic system models.

|  | **Only zones** | **Zones+**  **Subjects** | **Zones+**  **Medication** | **Zones+**  **Subjects+Medication** |
| --- | --- | --- | --- | --- |
| White-3 | 708 | 674 | 678 | 656 |
| Lover | 729 | 631 | 659 | 597 |
| White-5 | 624 | 585 | 573 | 546 |
| Macdonald | 614 | 579 | 573 | 548 |

Finally, we investigated the data for outliers. Figure A5 presents externally standardised residuals from the modified Macdonald system. From this figure we observe that most studies seem to be in good alignment with the incidence rates predicted for them, except in zone 8 (Monsoon Asia), there are three studies that could be interpreted potentially as outliers. These included one very large study (*n*=6393) with few patients who relapsed, and two small treatment studies with short follow up. We tried removing these data points, but this did not affect the model choice and there was no biological or methodological justification for doing so. Also, the model-based estimates of incidence rate were very similar to those shown in Table 2 in the main matter. In analogy to Figure A5, a forest plot (Figure A6) showed that the incidence rates in most studies were in a good alignment with the mean incidences in their respective geographic zones, but there were some cases where the incidences did not match. Thus, we concluded that the use of mixed-effects meta-analysis (which contains random effects attributable to individual studies), was warranted in this case. This is also observed in most studies of this type.

**Figures**


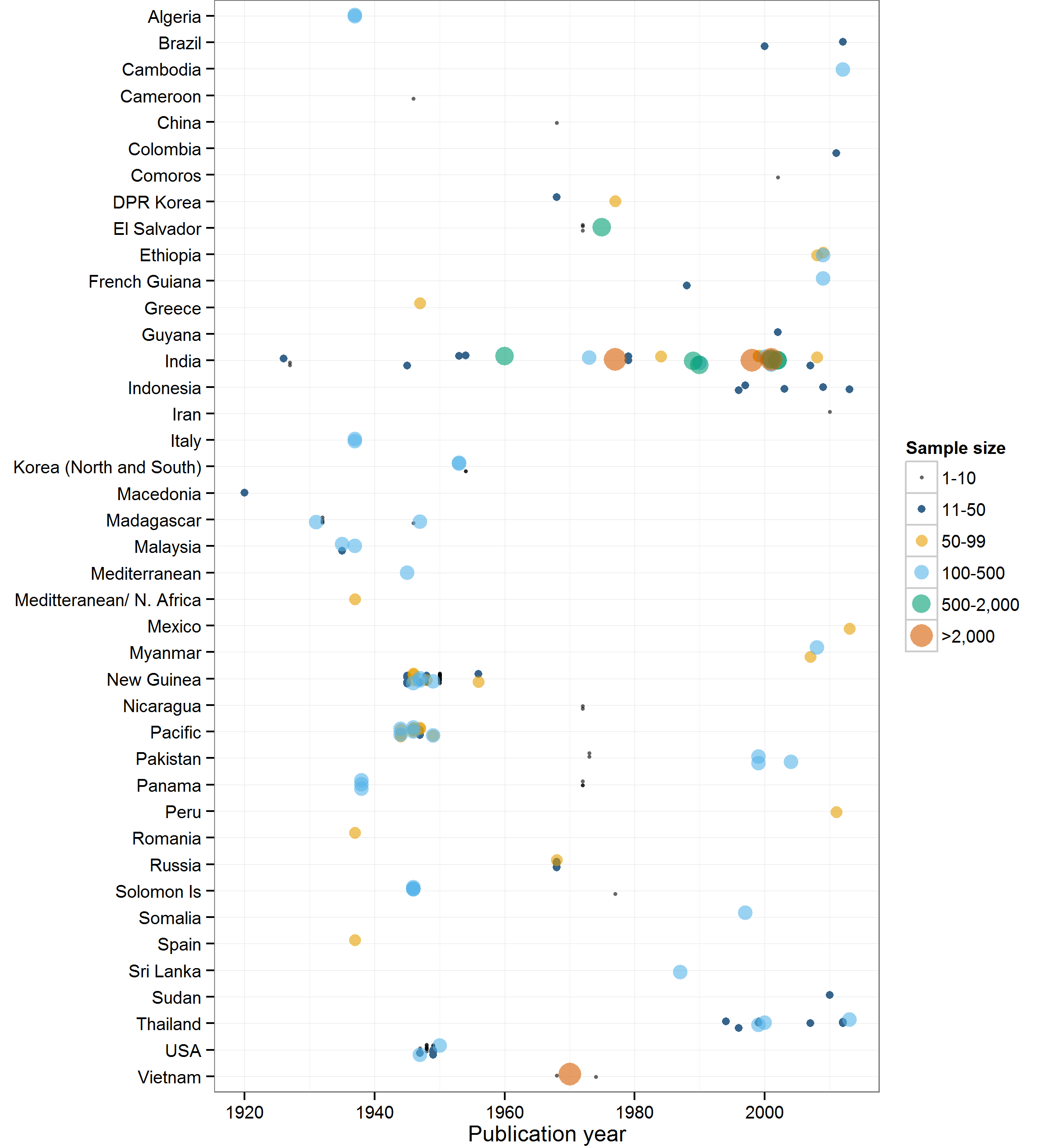


**Figure A1 Temporal distribution and size of studies per country or region**


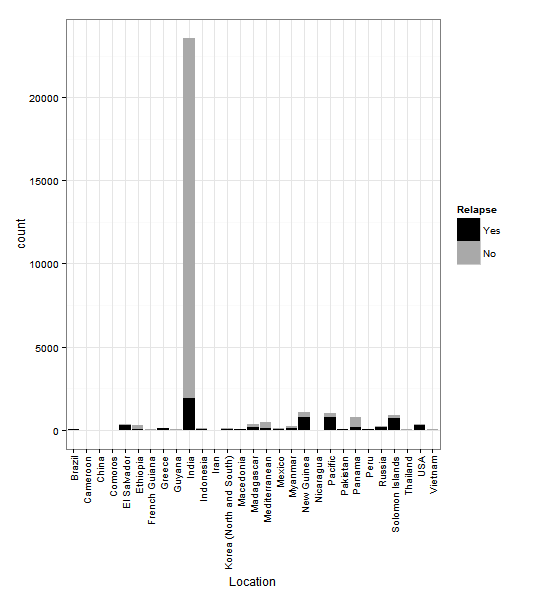


**Figure A2 Number of total cases and number of relapses observed per country or region**

**
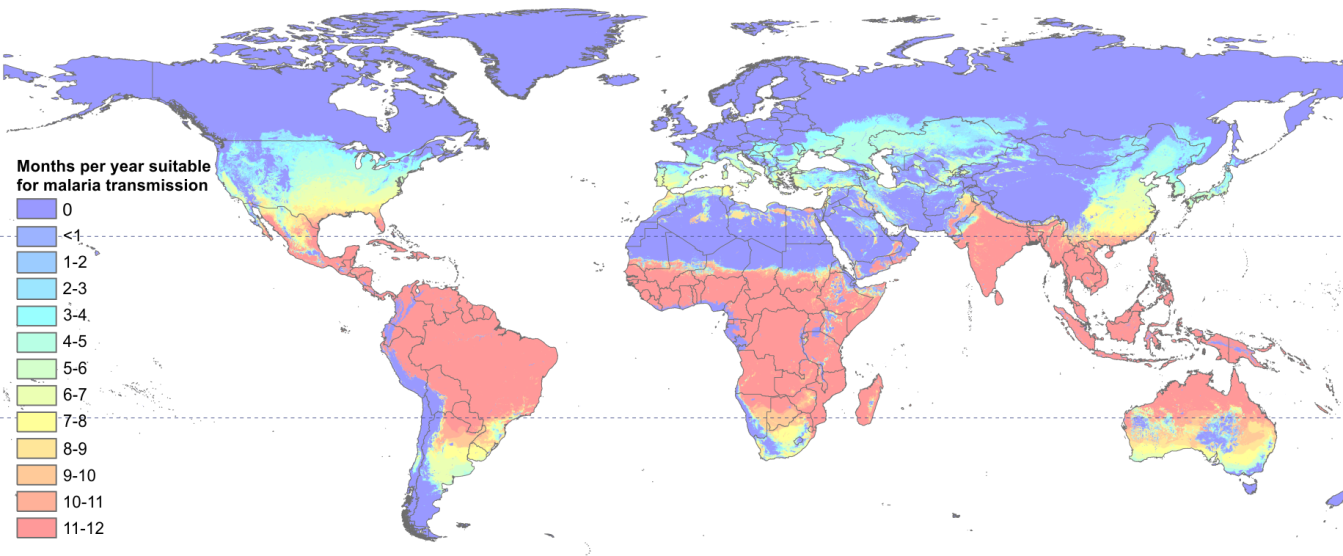
**

**Figure A3 Global variation in *Plasmodium vivax* transmission suitability**

The longest suitability period index classifies regions based on the number of months conditions are suitable for malaria transmission, ranging from year round (pink) to never suitable (blue). Values were calculated using monthly measures of *P. vivax* temperature suitability and EVI (see main text).


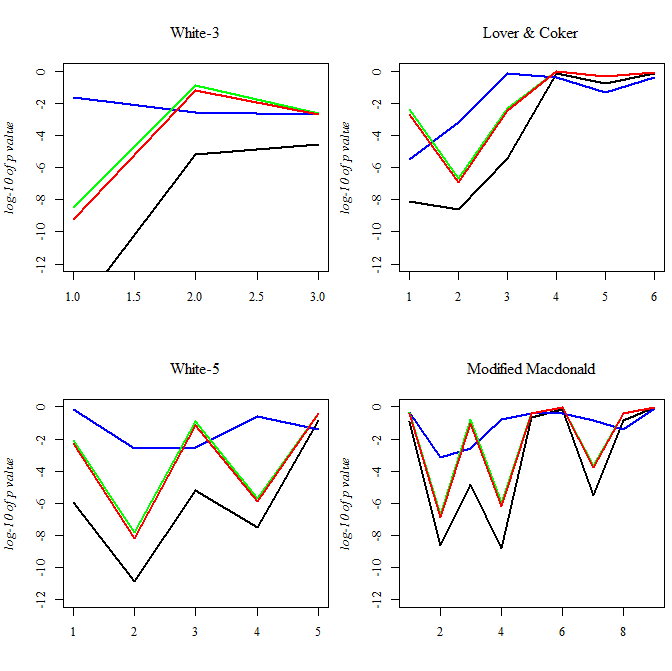


**Figure A4 Results of Shapiro-Wilk test for normality**

The Shapiro-Wilk test is a commonly used method for testing normality, which in turn is an underlying assumption of the meta-analysis models (i.e. R/metafor). Here we present the p values from this test. Points on the x-axes represent different geographic zones, while the lines themselves represent different transformations, such that logarithmic is blue, square root is green, Freeman-Tukey is red, and identity (no transformation) is black.


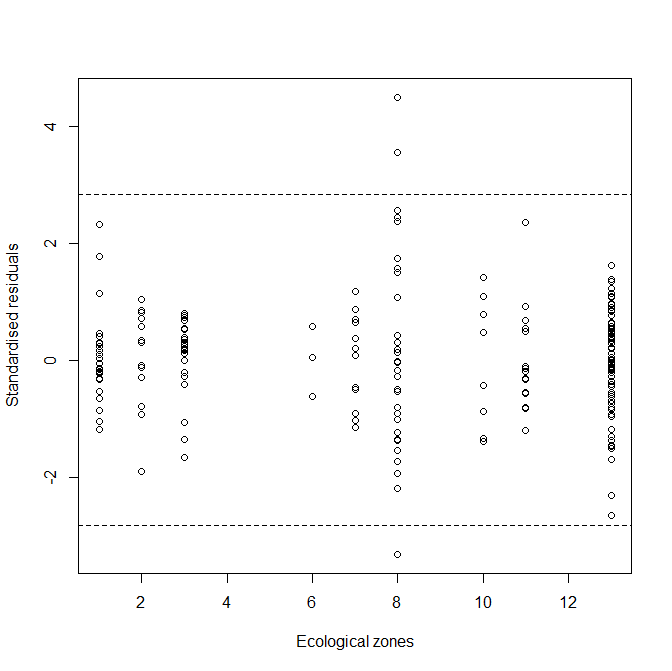
**Figure A5 Detecting outliers in the modified Macdonald system**

Each data point represents one study. The standardised residuals of the study effects are used here as a measure of model misfit. The horizontal lines are chosen such that there is *a priori* a 50 % chance that one of the residuals in a meta-analysis of this size falls outside these limits. Consequently, three of the studies could be interpreted as outliers.


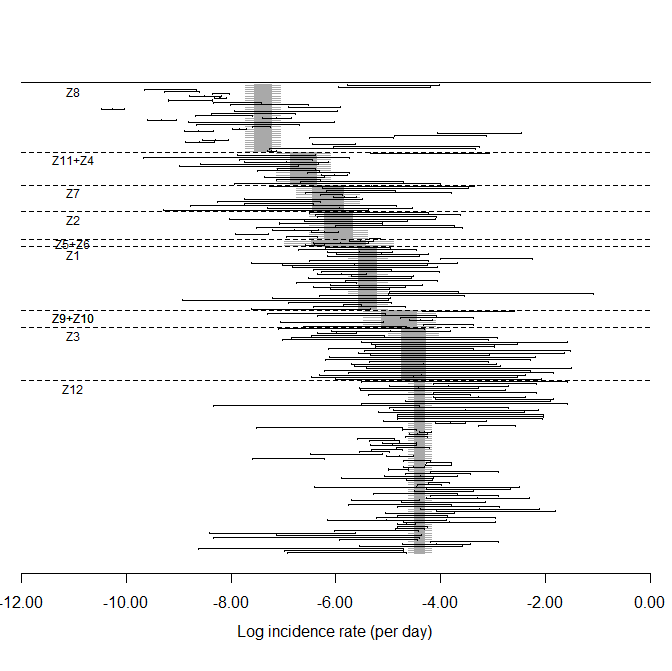


**Figure A6 Forest plot for the modified Macdonald system**

This figure has been produced by R/metafor . The x axis represents log-incidence rate, running from -12 to 0 with even spaces. The grey diamonds represent the confidence intervals of fixed effects, whereas the horizontal lines represent CIs of the incidence rate within each study. The two do not always overlap due to study-specific random effects. Note that the scale of the x axis is natural logarithm.

**References**

1. Gogtay NJ, Desai S, Kamtekar KD, Kadam VS, Dalvi SS, Kshirsagar NA: **Efficacies of 5- and 14-day primaquine regimens in the prevention of relapses in *Plasmodium vivax* infections.** *Ann Trop Med Parasitol* 1999, **93:**809-812.

2. Therneau T: **coxme: Mixed Effects Cox Models. R package version 2.2-3.** 2012.

3. Bates D, Maechler M, Bolker B, Walker S: **lme4: Linear mixed-effects models using Eigen and S4. R package version 1.0-5.** 2013.

4. Gething PW, Van Boeckel TP, Smith DL, Guerra CA, Patil AP, Snow RW, Hay SI: **Modelling the global constraints of temperature on transmission of *Plasmodium falciparum* and *P. vivax*.** *Parasit Vectors* 2011, **4:**92.

5. Viechtbauer W: **Conducting meta-analyses in R with the metafor package.** *J Stat Softw* 2010, **36:**1-48.
