# Supplementary material for: Geographical variation in Plasmodium vivax relapse
Source: Malar J. 2014 Apr 15;13:144. doi: 10.1186/1475-2875-13-144 (PMC4021508; doi:10.1186/1475-2875-13-144)
Supplement: Additional file 4 — Individual-level data reference list. [file 1475-2875-13-144-S4.docx]

**Additional File 4**

**Geographical variation in *Plasmodium vivax* relapse**

Authors: Katherine E Battle^1§^, Markku S Karhunen^1^, Samir Bhatt^1^, Peter W Gething^1^, Rosalind E Howes^1^, Nick Golding^1^, Thomas P Van Boeckel^2^, Jane P Messina^1^, G Dennis Shanks^3^, David L Smith^4,5^, J Kevin Baird^6.7^, Simon I Hay^1,5§^

^§^Authors for correspondence: katherine.battle@zoo.ox.ac.uk and simon.hay@zoo.ox.ac.uk

This file includes:

Individual-level relapse data literature references

**References listed in Additional File 3**

1. Adak T, Sharma VP, Orlov VS: **Studies on the *Plasmodium vivax r*elapse pattern in Delhi, India**. *Am J Trop Med Hyg* 1998, **59**(1):175-179.

2. Alving AS, Pullman TN, Craige B, Jones R, Whorton M, Eichelberger L: **The clinical trial of eighteen analogues of Pamaquin (Plasmochin) in vivax malaria, Chesson strain**. *J Clin Invest* 1948, **27**(3):34-45.

3. Appavoo NC, Roy RG, Kapali V: **Results of 3-day radical treatment of *Plasmodium vivax* in North Arcot and South Arcot Districts of Tamil Nadu**. *Indian J Malariol* 1984, **21**(1):21-24.

4. Arnold J, Alving AS, Hockwald RS, Clayman CB, Dern RJ, Beutler E: **Natural history of Korean vivax malaria after deliberate inoculation of human volunteers**. *J Lab Clin Med* 1954, **44**(5):723-726.

5. Baird JK, Leksana B, Masbar S, Fryauff DJ, Sutanihardja MA, Suradi, Wignall FS, Hoffman SL: **Diagnosis of resistance to chloroquine by *Plasmodium vivax*: timing of recurrence and whole blood chloroquine levels**. *Am J Trop Med Hyg* 1997, **56**(6):621-626.

6. Baird JK, Sustriayu Nalim MF, Basri H, Masbar S, Leksana B, Tjitra E, Dewi RM, Khairani M, Wignall FS: **Survey of resistance to chloroquine by *Plasmodium vivax* in Indonesia**. *Trans R Soc Trop Med Hyg* 1996, **90**(4):409-411.

7. Baird JK, Tiwari T, Martin GJ, Tamminga CL, Prout TM, Tjaden J, Bravet PP, Rawlins S, Ferrel M, Carucci D *et al*: **Chloroquine for the treatment of uncomplicated malaria in Guyana**. *Annals of Tropical Medicine & Parasitology* 2002, **96**(4):339-348.

8. Basavaraj HR: **Observations on the treatment of 678 malaria cases with primaquine in an area free from malaria transmission in Mysore State, India**. *Indian J Malariol* 1960, **14**(3):269-281.

9. Berliner RW, Earle DP, Taggart JV, Welch WJ, Zubrod CG, Knowlton P, Atchley JA, Shannon JA: **Studies on the chemotherapy of the human malarias. VII. The antimalarial activity of pamaquine**. *J Clin Invest* 1948, **27**(3 Pt 2):108-113.

10. Coatney GR, Cooper WC, et al.: **Studies in human malaria. VII. The protective and therapeutic action of quinine sulfate against St. Elizabeth strain vivax malaria**. *Am J Hyg* 1948, **47**(1):120-134.

11. Coatney GR, Cooper WC, Eyles DE, Culwell WB, White WC, Lints HA: **Studies in human malaria. XXVII. Observations on the use of pentaquine in the prevention and treatment of Chesson strain vivax malaria**. *J Natl Malar Soc* 1950, **9**(3):222-233.

12. Coatney GR, Cooper WC, Young MD: **Studies in human malaria. XXX. A summary of 204 sporozoite-induced infections with the Chesson strain of *Plasmodium vivax***. *J Natl Mal Soc* 1950, **9**(381-396).

13. Coatney GR, Cooper WC, Young MD, Burgess RW: **Studies in human malaria. IV. The suppressive action of a phenanthrene amino alcohol, NIH-204 (SN-1796) against sporozoite-induced vivax malaria (St. Elizabeth strain)**. *Am J Hyg* 1947, **46**(1):132-140.

14. Coatney GR, Cooper WC, Young MD, Burgess RW, Smarr RG: **Studies in human malaria. II. The suppressive action of sulfadiazine and sulfapyrazine against sporozoite-induced vivax malaria (St. Elizabeth strain)**. *Am J Hyg* 1947, **46**(1):105-118.

15. Coatney GR, Ruhe DS, et al.: **Studies in human malaria. X. The protective and therapeutic action of chloroquine (SN 7618) against St. Elizabeth strain vivax malaria**. *Am J Hyg* 1949, **49**(1):49-59.

16. Contacos PG, Collins WE, Jeffery GM, Krotoski WA, Howard WA: **Studies on the characterization of *Plasmodium vivax* strains from Central America**. *Am J Trop Med Hyg* 1972, **21**(5):707-712.

17. Contacos PG, Coatney GR, Collins WE, Briesch PE, Jeter MH: **Five day primaquine therapy--an evaluation of radical curative activity against vivax malaria infection**. *Am J Trop Med Hyg* 1973, **22**(6):693-695.

18. Contacos PG, Collins WE, Chin W, Jeter MH, Briesch PE: **Combined chloroquine-primaquine therapy against vivax malaria**. *Am J Trop Med Hyg* 1974, **23**(2):310-312.

19. Cooper WC, Coatney GR, Culwell WB, Eyles DE, Young MD: **Studies in human malaria. XXVI. Simultaneous infection with the Chesson and the St. Elizabeth strains of *Plasmodium vivax***. *J Natl Malar Soc* 1950, **9**(2):187-190.

20. Cooper WC, Coatney GR, Jeffery GM, Imboden CA, Jr.: **Studies in human malaria. XXVIII. Observations on the use of chloroguanide against the Chesson strain of *Plasmodium vivax***. *J Natl Malar Soc* 1950, **9**(4):366-376.

21. Cooper WC, Ruhe DS, Coatney CR, Josephson ES, Young MD: **Studies in human malaria. VIII. The protective and therapeutic action of quinacrine against St. Elizabeth strain vivax malaria**. *Am J Hyg* 1949, **49**(1):25-40.

22. de Araujo FC, de Rezende AM, Fontes CJ, Carvalho LH, Alves de Brito CF: **Multiple-clone activation of hypnozoites is the leading cause of relapse in *Plasmodium vivax* infection**. *PLoS One* 2012, **7**(11):e49871.

23. Downs WG: **Results in an infantry regiment of several plans of treatment for vivax malaria**. *Am J Trop Med Hyg* 1946, **26**:67-86.

24. Dua VK, Sharma VP: ***Plasmodium vivax* relapses after 5 days of primaquine treatment, in some industrial complexes of India**. *Annals of Tropical Medicine & Parasitology* 2001, **95**(7):655-659.

25. Gentzkow CJ, Callender GR: **Malaria in the the Panama Canal Department, United States Army II. results of treatment with quinine, atabrine and plasmochin**. *Am J Epidemiol* 1938, **28**(2):174-189.

26. Gogtay NJ, Desai S, Kamtekar KD, Kadam VS, Dalvi SS, Kshirsagar NA: **Efficacies of 5- and 14-day primaquine regimens in the prevention of relapses in *Plasmodium vivax* infections**. *Ann Trop Med Parasitol* 1999, **93**(8):809-812.

27. Gogtay NJ, Desai S, Kadam VS, Kamtekar KD, Dalvi SS, Kshirsagar NA: **Relapse pattern of *Plasmodium vivax* in Mumbai: a study of 283 cases of vivax malaria**. *J Assoc Physicians India* 2000, **48**(11):1085-1086.

28. Gonzalez-Ceron L, Mu J, Santillan F, Joy D, Sandoval MA, Camas G, Su X, Choy EV, Torreblanca R: **Molecular and epidemiological characterization of *Plasmodium vivax r*ecurrent infections in southern Mexico**. *Parasit Vectors* 2013, **6**(1):109.

29. Gordon HH, Dieuaide FR, Marble A, Christianson HB, Dahl LK: **Treatment of *Plasmodium vivax* malaria of foreign origin. A comparison of various drugs**. *Arch Intern Med* 1947, **79**(4):365-380.

30. Guthmann JP, Pittet A, Lesage A, Imwong M, Lindegardh N, Min Lwin M, Zaw T, Annerberg A, de Radigues X, Nosten F: ***Plasmodium vivax* resistance to chloroquine in Dawei, southern Myanmar**. *Trop Med Int Health* 2008, **13**(1):91-98.

31. Horing RO: **Induced and war malaria**. *J Trop Med Hyg* 1947, **50**(8):150-159.

32. Imboden CA, Jr., Cooper WC, Coatney GR, Jeffery GM: **Studies in human malaria. XXIX. Trials of aureomycin, chloramphenicol, penicillin, and dihydrostreptomycin against the Chesson strain of *Plasmodium vivax***. *J Natl Mal Soc* 1950, **9**(4):377-380.

33. James SP: **A malaria survey and some epidemiological observations in England**. In: *Malaria at Home and Abroad.* London: John Bale, Sons & Danielsson, LTD.; 1920: 80-93.

34. James SP: **Some general results of a study of induced malaria in England**. *Trans R Soc Trop Med Hyg* 1931, **24**(5):478-525.

35. Jeffery GM: **Relapses with Chesson strain *Plasmodium vivax* following treatment with chloroquine**. *Am J Trop Med Hyg* 1956, **5**(1):1-13.

36. Jiang JB, Huang JC, Liang DS, Liu JX, Zhang SW, Cheng FC: **Long incubation of *Plasmodium vivax multinucleatum* as demonstrated in three experimental human cases**. *Trans R Soc Trop Med Hyg* 1982, **76**(6):845-847.

37. Jones R, Jr., Pullman TN, et al.: **The therapeutic effectiveness of large doses of paludrine in acute attacks of sporozoite-induced vivax malaria, Chesson strain**. *J Clin Invest* 1948, **27**(3 Pt1):51-55.

38. Ketema T, Getahun K, Bacha K: **Therapeutic efficacy of chloroquine for treatment of *Plasmodium vivax* malaria cases in Halaba district, South Ethiopia**. *Parasit Vectors* 2011, **4**:46.

39. Lints HA, Coatney CR, Cooper WC, Culwell WB, White WC, Eyles DE: **Studies in human malaria. XXII. Prolonged suppression of Chesson strain vivax malaria by the weekly administration of chlorguanide or chloroquine**. *J Natl Mal Soc* 1950, **9**(1):50-58.

40. London IM, Lavietes PH, et al.: **The effects of quinacrine (atabrine) suppression on the course of Pacific vivax malaria**. *Am J Med* 1946, **1**(6):615-620.

41. Maisonneuve H, Joly F, John M, Carles G, Rossignol JF: **[Efficacy of halofantrine in *Plasmodium falciparum* or *Plasmodium vivax* malaria in a resistance area (French Guiana)]**. *Presse Med* 1988, **17**(3):99-102.

42. USA, MRU: **Malaria Report No. 306**. In: *Board for the Coordination of Malarial Studies: Relapse rates of benign tertian malaria following therapy.* Washington, D.C. and Oxford; 1944.

43. Alving AS: **Malaria Report No. 445**. In: *Board for the Coordination of Malarial Studies: Summary of clinical tests at stateville on 29 June 1945.* Washington, D.C.; 1945.

44. Alving AS: **Malaria Report No. 563**. In: *Board for the Coordination of Malarial Studies: Summar of clinical tests at Stateville on 13 December 1945* Washington, D.C.; 1945.

45. Alving AS: **Malaria Report No. 645**. In: *Board for the Coordination of Malarial Studies: Summary of clinical tests at Stateville.* Washington, D.C.; 1946.

46. Alving AS: **Malaria Report No. 672**. In: *Board for the Coordination of Malarial Studies: Summary of clinical tests at Stateville on 1 April 1946.* Washington, D.C.; 1946.

47. Alving AS: **Malaria Report No. 680**. In: *Board for the Coordination of Malarial Studies: Summary of clinical tests at Stateville on 1 May 1946.* Washington, D.C.; 1946.

48. Alving AS: **Malaria Report No. 702**. In: *Board for the Coordination of Malarial Studies: Summary of clinical tests at Stateville on 1 June 1946.* Washington, D.C.; 1946.

49. Mason J: **Patterns of *Plasmodium vivax* recurrence in a high-incidence coastal area of El Salvador, C. A**. *Am J Trop Med Hyg* 1975, **24**(4):581-585.

50. McLester JB: **Studies of relapse in malaria**. In: *Proceedings of the Conference of Army Physicians, Central Mediterranean Forces: 1945; Rome*: Institute Superiore di Sanita Viale Regina Marguerita; 1945: 4-7.

51. Most H, Kane CA, et al.: **Combined quinine-plasmochin treatment of vivax malaria; effect of relapse rate**. *Am J Med Sci* 1946, **212**(5):550-560.

52. Most H, London IM, Kane CA, Lavietes PH, Schroeder EF, Hayman JM: **Chroloquine for treatment of acute attacks fo vivax-malaria**. *JAMA* 1946, **131**(12):963-967.

53. Nateghpour M, Mavi SA, Keshavarz H, Rezaei S, Abedi F, Edrissian G, Raeisi A: **Molecular monitoring of *Plasmodium vivax* infection after radical treatment in southeastern Iran**. *Iran J Arthropod Borne Dis* 2010, **4**(1):24-30.

54. Pukrittayakamee S, Vanijanonta S, Chantra A, Clemens R, White NJ: **Blood stage antimalarial efficacy of primaquine in *Plasmodium vivax* malaria**. *J Infect Dis* 1994, **169**(4):932-935.

55. Pullman TN, Craige B, Jr., et al.: **Comparison of chloroquine, quinacrine (atabrine) and quinine in the treatment of acute attacks of sporozoite-induced vivax malaria, Chesson strain**. *J Clin Invest* 1948, **27**(3 Pt1):46-50.

56. Ranque S, Badiaga S, Delmont J, Brouqui P: **Triangular test applied to the clinical trial of azithromycin against relapses in *Plasmodium vivax* infections**. *Malar J* 2002, **1**:13.

57. Roy RG, Chakrapani KP, Dhinagaran D, Sitaraman NL, Ghosh RB: **Efficacy of 5-day radical treatment of *P. vivax* infection in Tamil Nadu**. *Indian J Med Res* 1977, **65**(5):652-656.

58. Roy RG, Shanmugham CA, Chakrapani KP, Ganesan AV: **Results of 5-day course of radical treatment of *Plasmodium vivax* in six districts of Tamil Nadu**. *Indian J Med Res* 1979, **69**:939-943.

59. Ruhe DS, Cooper WC, Coatney GR, Josephson ES, Young MD: **Studies in human malaria: IX. The protective and therapeutic action of SN 6911 (Sontochin) against St. Elizabeth stain vivax malaria**. *Am J Hyg* 1949, **49**:41-48.

60. Ruhe DS, Cooper WC, Coatney CR, Josephson ES: **Studies in human malaria. XII. The protective and therapeutic action of SN 5241 against St. Elizabeth strain vivax malaria**. *Am J Hyg* 1949, **49**(3):346-354.

61. Ruhe DS, Cooper WC, Coatney GR, Josephson ES: **Studies in human malaria: XIV. The ineffectiveness of Colchicine (SN 12,080), SN 7266 and SN 8557 as curative agents against St. Elizabeth strain vivax malaria**. *Am J Hyg* 1949, **49**:361-366.

62. Saint-Yves IF: **Comparison of treatment schedules for *Plasmodium vivax* infections in the Solomon Islands**. *P N G Med J* 1977, **20**(2):62-65.

63. Shannon JA: **Rationale underlying the clinical evaluation of antimalarial drugs**. In: *A Survey of Antimalarial Drugs: 1941–1945.* Edited by Wiselogle FY. Ann Arbor, Michigan: J.W. Edwards; 1946: 177-220.

64. Shannon JA, Earle DP, Berliner RW, Taggart JV: **Studies on the chemotherapy of the human malarias. I. Method for the quantitative assay of suppressive antimalarial action in vivax malaria**. *J Clin Invest* 1948, **27**(3):66-74.

65. Sharma MID, Sehgal PN, Vaid BK, Dubey RC, Nagendra S, Paithne PK, Joshi ML: **Effectiveness of drug schedule being followed under the National Malaria Eradication Programme, India, for radical cure of vivax malaria cases**. *J Commun Dis* 1973, **5**(4):167-174.

66. Sharma RC, Gautam AS, Orlov V, Sharma VP: **Relapse pattern of *Plasmodium vivax* in Kheda district, Gujarat**. *Indian J Malariol* 1990, **27**(2):95-99.

67. Shute PG: **Latency and long-term relapses in benign tertian malaria**. *Trans R Soc Trop Med Hyg* 1946, **40**(2):189-200.

68. Singh J, Ray AP, Basu PC, Nair CP: **Preliminary studies on 8-aminoquinolines**. *Indian J Malariol* 1953, **7**(3):289-294.

69. Singh J, Ray AP, Misra BG, Nair CP: **Antirelapse treatment with primaquine and pyrimethamine**. *Indian J Malariol* 1954, **8**(2):127-136.

70. Singh N, Mishra AK, Sharma VP: **Radical treatment of vivax malaria in Madhya Pradesh, India**. *Indian J Malariol* 1990, **27**(1):55-56.

71. Sinha S, Dua VK, Sharma VP: **Efficacy of 5 day radical treatment of primaquine in *Plasmodium vivax* cases at the BHEL industrial complex, Hardwar (U.P.)**. *Indian J Malariol* 1989, **26**(2):83-86.

72. Sinton JA: **Studies in malaria with special reference to treatment. VII. The intravenous injection of sodium stovarsol in the treatment of benign tertian malaria**. *Indian J Med Res* 1927, **15**:287-299.

73. Srivastava HC, Yadav RS, Joshi H, Valecha N, Mallick PK, Prajapati SK, Dash AP: **Therapeutic responses of *Plasmodium vivax* and *P. falciparum* to chloroquine, in an area of western India where *P. vivax* predominates**. *Ann Trop Med Parasitol* 2008, **102**(6):471-480.

74. Sumawinata IW, Bernadeta, Leksana B, Sutamihardja A, Purnomo, Subianto B, Sekartuti, Fryauff DJ, Baird JK: **Very high risk of therapeutic failure with chloroquine for uncomplicated *Plasmodium falciparum* and *P. vivax* malaria in Indonesian Papua**. *Am J Trop Med Hyg* 2003, **68**(4):416-420.

75. Sutanto I, Suprijanto S, Nurhayati, Manoempil P, Baird JK: **Resistance to chloroquine by *Plasmodium vivax* at Alor in the Lesser Sundas Archipelago in eastern Indonesia**. *Am J Trop Med Hyg* 2009, **81**(2):338-342.

76. Swellengrebel NH, De Buck A: **Plasmoquine prophylaxis in benign tertian malaria**. *Proc Koninklijke Nederlandse Akademie Wetenschappen* 1932, **35**:912-914.

77. Teka H, Petros B, Yamuah L, Tesfaye G, Elhassan I, Muchohi S, Kokwaro G, Aseffa A, Engers H: **Chloroquine-resistant *Plasmodium vivax* malaria in Debre Zeit, Ethiopia**. *Malar J* 2008, **7**:220.

78. Tiburskaja NA, Vrublevskaja OS: **The course of infection caused by the North Korean strain of *Plasmodium vivax***. *WHO/MAL/77* 1977, **895**:1-19.

79. Tiburskaja NA, Sergiev PG, Vrublevskaja OS: **Dates of onset of relapses and the duration of infection in induced tertian malaria with short and long incubation periods**. *Bull World Health Organ* 1968, **38**(3):447-457.

80. Trager W, Bang FB, Hairston NG: **The effect of four different therapies on the relapse rate of vivax malaria**. *Am J Hyg* 1947, **45**(1):43-57.

81. Van den Eede P, Soto-Calle VE, Delgado C, Gamboa D, Grande T, Rodriguez H, Llanos-Cuentas A, Anne J, D'Alessandro U, Erhart A: ***Plasmodium vivax* sub-patent infections after radical treatment are common in Peruvian patients: results of a 1-year prospective cohort study**. *PLoS One* 2011, **6**(1):e16257.

82. Villalobos-Salcedo JM, Tada MS, Kimura E, Menezes MJ, Pereira da Silva LH: **In-vivo sensitivity of *Plasmodium vivax* isolates from Rondonia (western Amazon region, Brazil) to regimens including chloroquine and primaquine**. *Ann Trop Med Parasitol* 2000, **94**(8):749-758.

83. Walsh DS, Looareesuwan S, Wilairatana P, Heppner DG, Jr., Tang DB, Brewer TG, Chokejindachai W, Viriyavejakul P, Kyle DE, Milhous WK *et al*: **Randomized dose-ranging study of the safety and efficacy of WR 238605 (Tafenoquine) in the prevention of relapse of *Plasmodium vivax* malaria in Thailand**. *J Infect Dis* 1999, **180**(4):1282-1287.

84. White WC, Cooper WC, et al.: **Studies in human malaria. XXI. The cure of St. Elizabeth strain vivax malaria by pentaquine-quinine, administered during acute attacks or during latency**. *J Natl Malar Soc* 1948, **7**(4):316-321.

85. Yadav RS, Ghosh SK: **Radical curative efficacy of five-day regimen of primaquine for treatment of *Plasmodium vivax* malaria in India**. *J Parasitol* 2002, **88**(5):1042-1044.

86. Yeshiwondim AK, Tekle AH, Dengela DO, Yohannes AM, Teklehaimanot A: **Therapeutic efficacy of chloroquine and chloroquine plus primaquine for the treatment of *Plasmodium vivax* in Ethiopia**. *Acta Trop* 2010, **113**(2):105-113.

87. Yorke W: **Further observations on malaria made during treatment of general paralysis**. *Trans R Soc Trop Med Hyg* 1926, **19**(3):108-131.
